# Spatially localised expression of the glutamate decarboxylase *gadB* in *Escherichia coli* O157:H7 microcolonies in hydrogel matrices

**DOI:** 10.1038/s41538-023-00229-8

**Published:** 2023-10-14

**Authors:** Cédric Saint Martin, Nelly Caccia, Maud Darsonval, Marina Gregoire, Arthur Combeau, Grégory Jubelin, Florence Dubois-Brissonnet, Sabine Leroy, Romain Briandet, Mickaël Desvaux

**Affiliations:** 1 Université Paris-Saclay, INRAE, AgroParisTech, MICALIS Institute, 78350 Jouy-en-Josas, France; 2 INRAE, UCA, UMR0454 MEDIS, 63000 Clermont-Ferrand, France

**Keywords:** Bacteria, Applied microbiology, Bacteriology, Pathogens

## Abstract

Functional diversity within isogenic spatially organised bacterial populations has been shown to trigger emergent community properties such as stress tolerance. Considering *gadB* gene encoding a key glutamate decarboxylase involved in *E. coli* tolerance to acidic conditions, we investigated its expression in hydrogels mimicking the texture of some structured food matrices (such as minced meat or soft cheese). Taking advantage of confocal laser scanning microscopy combined with a genetically-engineered dual fluorescent reporter system, it was possible to visualise the spatial patterns of bacterial gene expression from in-gel microcolonies. In *E. coli* O157:H7 microcolonies, *gadB* showed radically different expression patterns between neutral (pH 7) or acidic (pH 5) hydrogels. Differential spatial expression was determined in acidic hydrogels with a strong expression of *gadB* at the microcolony periphery. Strikingly, very similar spatial patterns of *gadB* expression were further observed for *E. coli* O157:H7 grown in the presence of *L. lactis*. Considering the ingestion of contaminated foodstuff, survival of *E. coli* O157:H7 to acidic stomachal stress (pH 2) was significantly increased for bacterial cells grown in microcolonies in acidic hydrogels compared to planktonic cells. These findings have significant implications for risk assessment and public health as they highlight inherent differences in bacterial physiology and virulence between liquid and structured food products. The contrasting characteristics observed underscore the need to consider the distinct challenges posed by these food types, thereby emphasising the importance of tailored risk mitigation strategies.

## Introduction

*Escherichia coli* is a commensal bacterium found in the gut of mammals that plays an integral part in the digestive process. However, some strains of *E. coli* are pathogenic and represent a public health issue when they reach the production chain lines in food industries. Shigatoxin (Stx) encoding *E. coli* (STEC) are the third most common foodborne zoonosis in Europe^1^ and amongst STEC, the serotype O157:H7 is commonly identified in patients. *E. coli* O157:H7 are enterohaemorrhagic *E. coli* (EHEC) responsible for bloody diarrhoea when the intestinal lining is broken by the presence of Stx. A possible outcome of Stx passing in the bloodstream is damage to the kidneys that can lead to a haemolytic uraemic syndrome (HUS), which itself lead to fatal outcomes in 5% of cases^2^. Children are especially at risk and *E. coli* O157:H7 is still a main cause of paediatric HUS^3^. Excepting evisceration accidents where carcasses are excluded from the production chain line, food contamination of animal products (meat and milk products), vegetable or water can occur through direct or indirect faecal contamination by STEC^4–6^. Storage and holding temperatures may also influence *E. coli* O157:H7 survival in food products^7^. At the level of the European Union (EU), regulations ask for the absence of this pathogen in 25 g of germinated seeds (Regulation CE 209/2013, amendment 2073/2055), but no equivalent exist for meat or milk products. Though, precautionary measures exist at the state level in the EU (France, DGAL/SSDSA/2016-353, DGAL/SDSSA/2018-9). However, this pathogen is still routinely detected at levels above 100 CFU/g in more than 1% of all red meats, which remain a main vector of infection by EHEC in EU^1,8^.

Structured food matrices present heterogeneous local microenvironments harbouring multiple micro-gradients that can evolve with time and microbial activity^9^. This leads bacterial cells in food matrices to face different biotopes in which their growth and behaviour can diverge from observations in liquid laboratory media. Therefore, environmental conditions of food matrices can prompt high phenotypic diversity in microbial populations as the cells adapt to local microenvironments^10,11^. In comparison with their planktonic counterparts, phenotypic diversity in structured communities can influence bacterial fitness and behaviour, such as increase expression of virulence genes^12^, higher tolerance to antimicrobials agents and thermal stress^13,14^, or improved cell motility^15^. While several studies reported emergent properties of bacterial community in food matrices at the population level^13,16–19^, no experimental evidence has yet been reported on the spatial heterogeneity of gene expression at the scale of single cells.

The stomachal phase after food ingestion exposes bacteria to strong acidic pH conditions for several hours and is credited for the highest population reduction of the bacterial load. High tolerance to acidic conditions is therefore necessary for foodborne pathogens, and involved systems that regulate intracellular pH. The glutamic acid decarboxylase (GAD) is a common system of acid resistance (AR) found in bacteria able to survive in extreme acid conditions^20–24^. In *E. coli*, the GAD system is a three components system: two glutamate decarboxylases, GadA and GadB, which use cytoplasmic free protons by converting glutamate into γ-aminobutyrate (GABA), and the glutamate/GABA antiporter GadC. When the pH is below 5.6, cytoplasmic GadB migrates near the inner membrane to maximise collaboration with transmembrane GadC^25^. While *gadA* is independent in chromosomic location and *gadB* and *gadC* are organised in operon, the expression of both *gadA* and *gadBC* is transcriptionally regulated by RpoS, two AraC-like regulators GadX and GadW, and by effectors with two inhibitors, the cyclic AMP receptor protein and H-NS. H-NS and RpoS in particular determine the temporal expression, the former inhibiting *gadB* expression, whereas RpoS promotes the transcription of *gadB* once the stationary phase is reached^26^.

To decipher and model fitness and behaviour of *E. coli* O157:H7, synthetic microbial ecology approaches were used in structured food matrices where the complexity of the communities and the factors of influence are reduced to their minimum, but increased in their controllability^27^. Such approaches have been used to describe how matrix parameters affect bacterial growth and morphodynamics of microcolonies^16,28^. In a recent contribution, we have shown that the volume, distribution and sphericity of microcolonies of *E. coli* O157:H7 in hydrogels are dependent of the size of the inoculum, but also on the concentration of acids and NaCl, two environmental stresses frequently encountered in food products^29^.

In this study, we took advantage of hydrogels to observe the local expression of *gadB* in *E. coli* O157:H7 cells in microcolonies using confocal laser scanning microscopy (CLSM). To explore the expression patterns in microcolonies, a dual fluorescent reporter system was engineered to monitor the spatial expression of *gadB* at the single-cell scale. In order to relate the impact that phenotypic heterogeneity in microcolonies can have on community function, the survival of planktonically grown cells to a strongly acidic media mimicking the stomachal passage was further assessed and compared to cells grown or dispersed in hydrogels.

## Results

### Spatial patterns of *gadB* expression from in-gel microcolonies of *E. coli* O157:H7

**Fig. 1:**
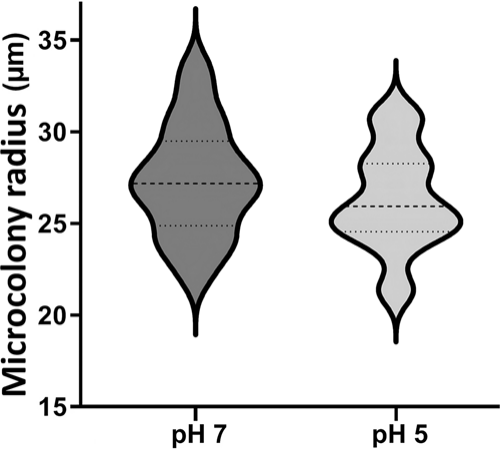
Violin plot of the radius of *E. coli* O157:H7 microcolonies in neutral (pH 7) or acidic (pH 5) hydrogels. In-gel microcolonies were observed in neutral LMPA matrix (pH 7) or in acid LMPA matrix (pH 5). The radius of the microcolonies is expressed in µm, where the width of each figure represents the concentration of the number of values. For each case, the dashed line is the mean value of radius, and dotted lines delimit the 75% probability interval. Radius values were calculated from 40 independent microcolonies. LMPA low melting point agarose.

**Fig. 2:**
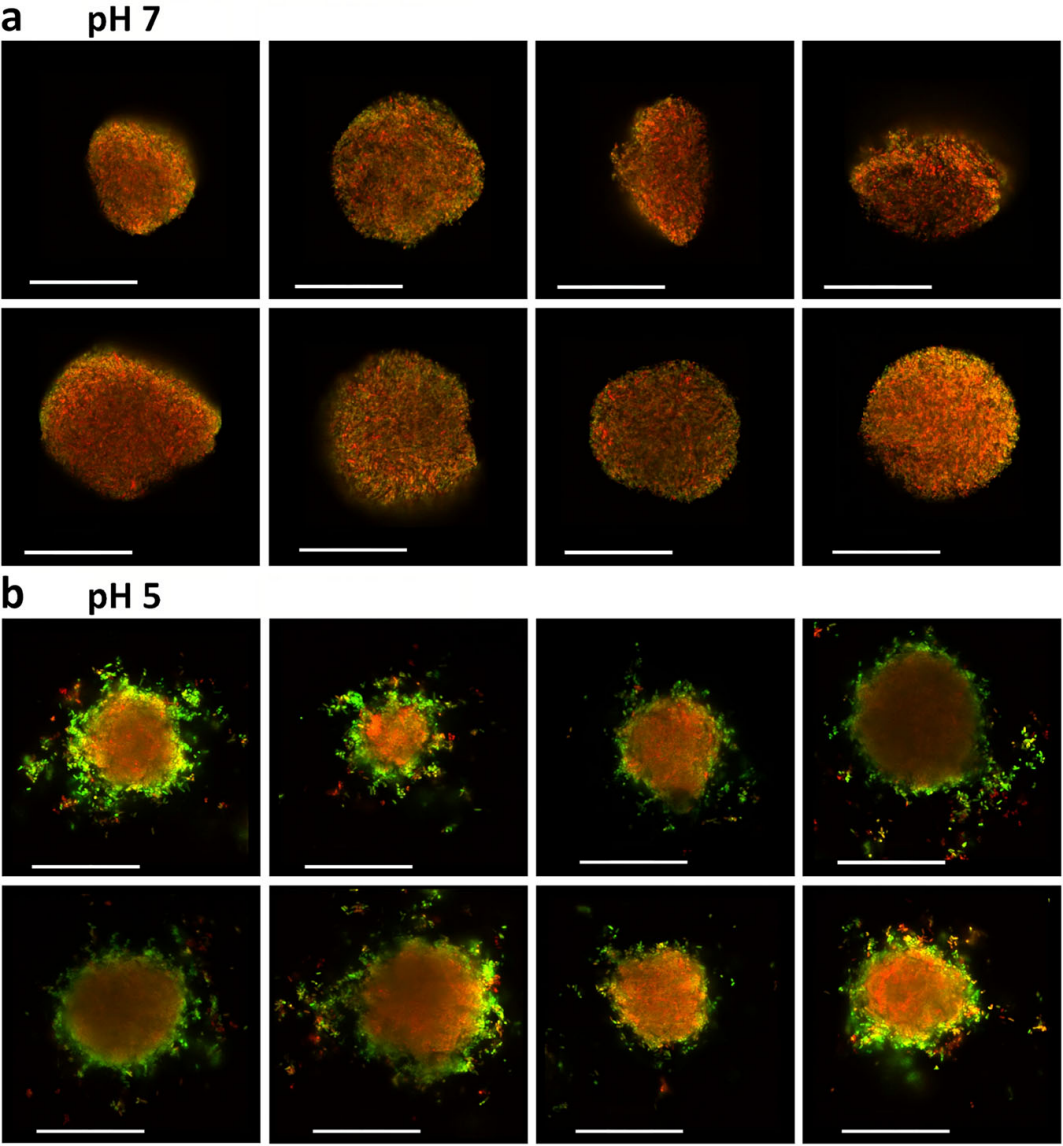
Representative images of *gadB* spatial expression patterns from *E. coli* O157:H7 microcolonies cultivated in neutral (pH 7) or acidic (pH 5) hydrogels. Microscopic examination of eight representative in-gel microcolonies either in **a** neutral LMPA matrix (pH 7) or **b** in acid LMPA matrix (pH 5) from 5-µm-thick slices. The red fluorescence is constitutive and the green fluorescence is expressed as a function of *gadB* expression. Length of the scale bars represents 50 µm. LMPA low melting point agarose.

To observe in-gel microcolonies of *E. coli* O157:H7 and investigate *gadB* expression, two hydrogels were considered using neutral (pH 7) or acid (pH 5) agarose matrices. There were no statistically significant differences observed in the mean radius of microcolonies grown in neutral and acidic hydrogels (27 and 28 µm, respectively), as determined from measurements on 40 independent microcolonies (*P* > 0.05; Fig. 1). However, at pH 5, microcolonies appear more circular than at pH 7 and harbour a more disperse layer at their edge shedding from the colony core, forming a crown around it (Fig. 2).

**Fig. 3:**
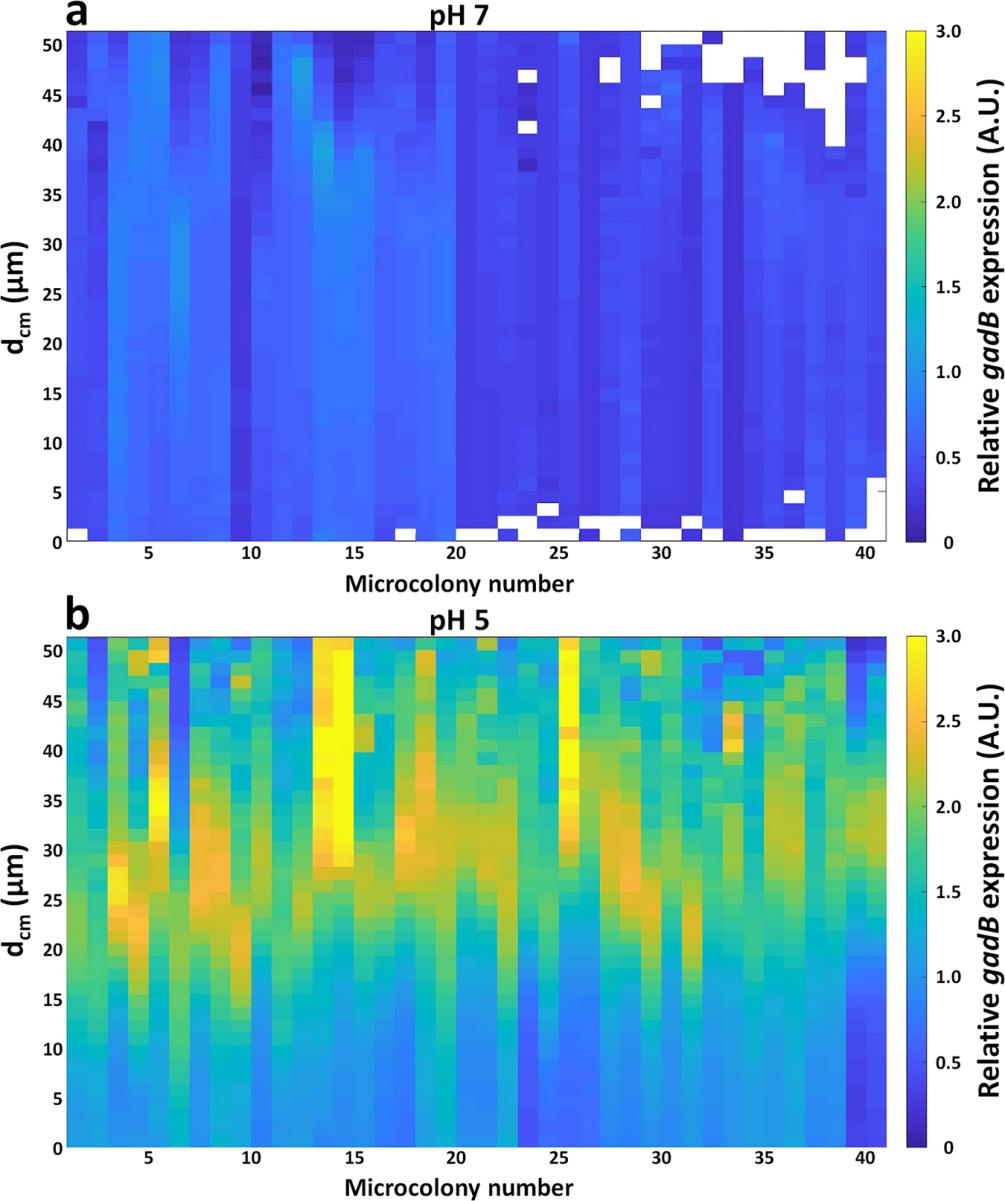
Relative expression of *gadB* in *E. coli* O157:H7 microcolonies as a function of the distance from the centre of microcolony in neutral (pH 7) or acidic (pH 5) hydrogels. Kymograms of the relative *gadB* expression provided as the ratio of green fluorescence intensity over red fluorescent intensity (GFP reporter/RFP constitutive) as a function of the distance from the centre of the microcolony (*d*_cm_) in **a** neutral LMPA matrix (pH 7) or **b** in acid LMPA matrix (pH 5). Data results from 40 independent microcolonies. LMPA low melting point agarose, GFP green fluorescent protein, RFP red fluorescent protein.

Microscopic observations of the fluorescence reporting the expression of *gadB* in cells inside microcolonies (Fig. 2) show radically different patterns between the two hydrogels. In neutral pH conditions, the gene is expressed at a basal level throughout the whole microcolony with no specific spatial arrangement (Fig. 2a and Supplementary Fig. 4). By contrast, the expression of *gadB* is strongly expressed at the periphery of the microcolonies formed in acidic hydrogels (Fig. 2b and Supplementary Fig. 4). Those qualitative observations were reinforced by a quantitative exploration of the radial distribution of *gadB* expression (Fig. 3). For both agarose matrices (neutral and acid conditions), the genetic expression is monitored by the green fluorescent intensity normalised with the red constitutive fluorescent intensity. 3D kymographs integrating 40 independent microcolonies (*X*-axis) for each condition represent in colour code *gadB* transcription from the centre of the microcolony (*Y*-axis, *d*_CM_ = 0 µm) to its edges and beyond. In neutral hydrogel, *gadB* expression is low and almost constant over the radius of the microcolonies (Fig. 3a), but in acidic hydrogels, the microcolonies present a sharp band of *gadB* strong expression between 25 and 30 µm from the centre of the microcolonies (Fig. 3b). This is consistent with the observed spatial expression as the radius of the microcolonies is 27–28 µm (±5 µm) in these experimental conditions. Interestingly, when microcolonies merge as they grow, they behave like a single colony in regard to the peripheral *gadB* spatial expression. Similarly, if two microcolonies are in near contact, the two sides facing each other do not present a strong expression of *gadB* or the shedding of single cells visible in other areas at the periphery (Supplementary Fig. 5).

**Fig. 4:**
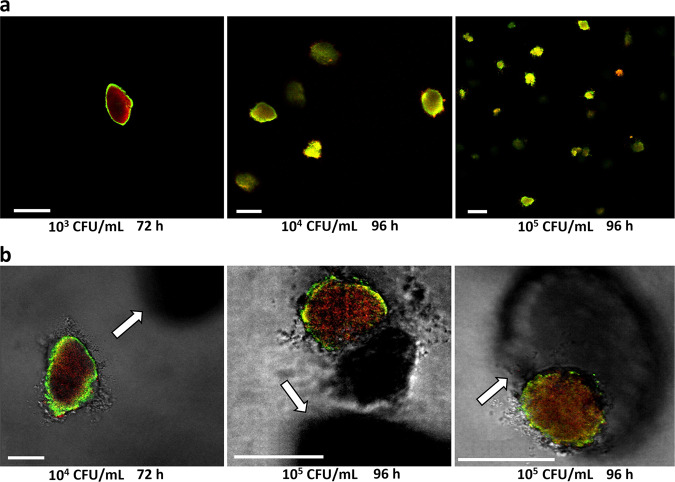
Spatial patterns of expression of *gadB* for *E. coli* O157:H7 in the presence of *L. lactis*, cultivated in neutral (pH 7) hydrogels. **a** Representative microscopic observations of in-gel microcolonies of *E. coli* O157:H7 co-inoculated (at 10^3^, 10^4^ or 10^5^ CFU/ml) with *L. lactis* (inoculated at 10^3^ CFU/ml), in neutral LMPA matrix (pH 7). The red fluorescence is constitutive and the green fluorescence is expressed as a function of *gadB* expression. Length of the scale bars represents 100 µm. **b** Focus on microcolonies of *E. coli* O157:H7 close or in contact with *L. lactis* microcolonies as observed by fluorescence and transmitted light from 5-µm-thick slices. In each panel A and B, the first picture (on the left) was taken at 72 h, whereas the second and third pictures (middle and right) were taken at 96 h. *L. lactis* microcolonies are visualised in the bottom images thanks to the transmission detection (indicated by white arrows). Length of the scale bars represents 50 µm. LMPA low melting point agarose, CFU colony forming unit.

Considering its recognised ability to produce lactic acid, *E. coli* O157:H7 was then cultured in the presence of *L. lactis* ssp*. cremoris* (Fig. 4). As previously observed^29^, the initial load of the *E. coli* O157:H7 inoculum influences the size of the microcolonies, which possess the same morphology as seen in mono-cultures in the acidic condition (Fig. 4a). From 50 µm thick slices, close up on *E. coli* O157:H7 microcolonies in adjacent proximity to microcolonies of *L. lactis* clearly shows the same differential spatial patterns of *gadB* expression as previously observed for the mono-cultures in acidic conditions (Fig. 4b and Supplementary Fig. 7A).

### Increased survival to acidic stomachal stress of *E. coli* O157:H7 grown in gel-microcolonies

**Fig. 5:**
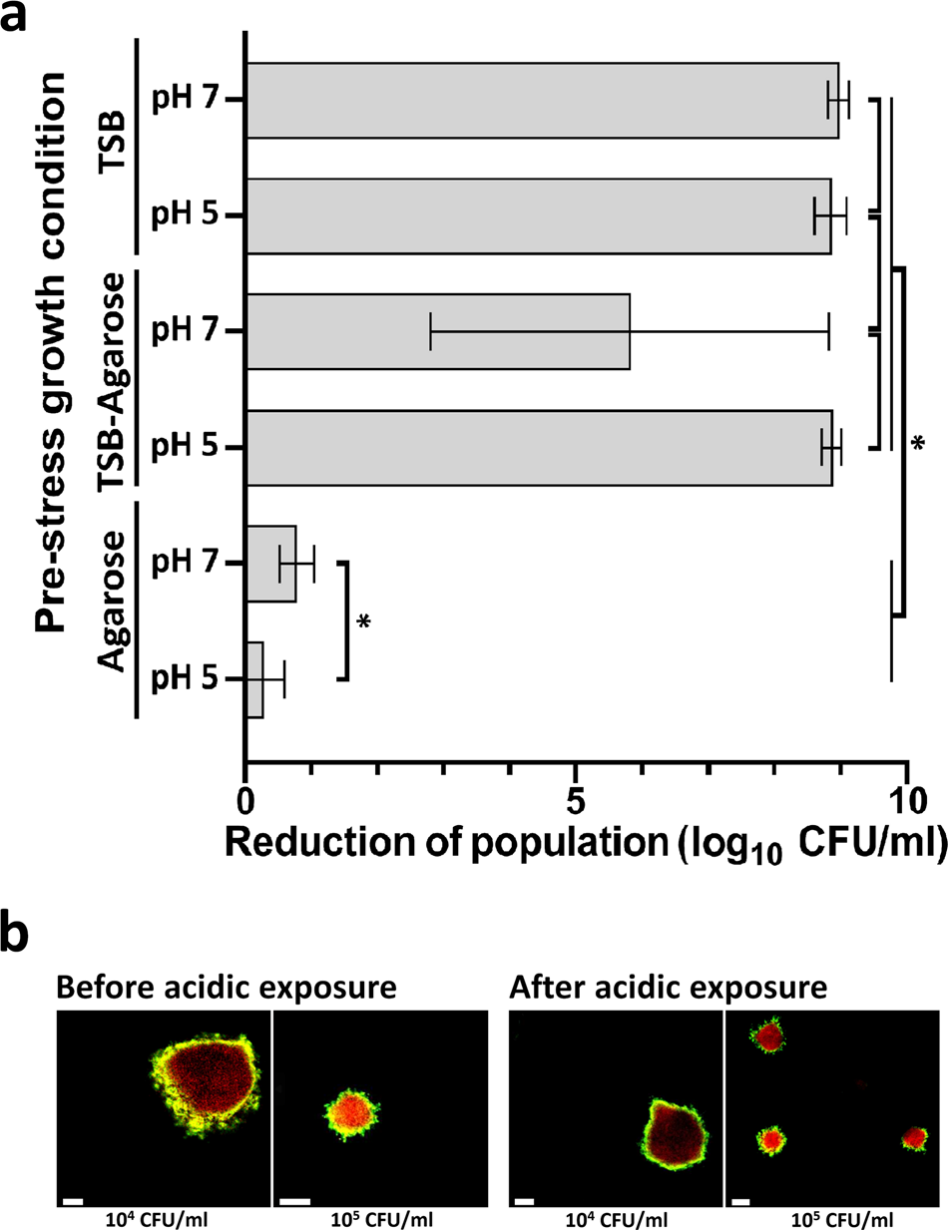
Population log-reduction of planktonic cultures and hydrogel microcolonies of *E. coli* O157:H7 upon acidic exposure (pH 2). Bacterial cells were exposed to HCl pH 2 for 4 h. **a** Representation of the mean reduction of population log between the control (before acidic exposure) and survival (after acidic exposure) groups. From the top of the picture, bacteria incubated and tested in planktonic conditions (liquid) are shown in the first and second bar (TSB), those grown as planktonic cells and encased in agarose before testing are shown in the third and fourth bar (TSB-Agarose), and the results for populations incubated and tested in LMPA matrices (Agarose) are shown in the fifth and sixth bar (Agarose). A star indicates a significant difference between values (*P* < 0.05). Data resulted from at least six biological replicates and statistically tested following an ANOVA (analysis of variance). Error bars correspond standard deviation (s.d.). **b** Representative microscopic observations of in-gel microcolonies of *E. coli* O157*:*H7 incubated and tested in LMPA matrices (Agarose pH 5) before and after acidic exposure. Length of the scale bars represents 50 µm. LMPA low melting point agarose, TSB tryptone soya broth, CFU colony forming unit.

As the capacity of survival to stomachal acidic stress of *E. coli* O157:H7 populations is of interest for public health safety, the acid resistance of bacteria grown planktonically or in hydrogel matrices was evaluated by enumeration on agar after acidic exposure (HCl pH 2). Cultures of *E. coli* O157:H7 adjusted to 10^4^ CFU/ml were incubated at neutral (pH 7) or acid pH (pH 5) in either tryptone soya broth (TSB; liquid) or agarose matrices (Fig. 5). After 96 h of incubation at 20°C, the populations reached values of log_10_ CFU/ml of 9.4 and 9.6 in TSB at neutral and acid pH, respectively, and of 9.5 and 8.6 in agarose matrices (at neutral and acid pH, respectively) (Fig. 5a). Upon acidic exposure, in-gel microcolonies of *E. coli* O157:H7 incubated and tested in agarose matrices show differential spatial expression patterns for *gadB* (Fig. 5b). Of note, *E. coli* O157:H7 microcolonies grown in the presence of *L. lactis* also show similar differential spatial expression patterns for *gadB* after acidic exposure (Supplementary Fig. 7B).

Bacteria grown in planktonic conditions in TSB were highly sensitive to the 4-h exposition at pH 2 with a total loss of the culturable population (9 log reduction) (Fig. 5a). In contrast, cells grown as spatially organised microcolonies in agarose matrices for 96 h presented a statistically high tolerance to this strong acidic stress (*P* < 0.05). The best tolerance was observed for the microcolonies incubated at pH 5 with a log reduction as low as 0.29 log CFU/ml, statistically significantly lower than the reduction observed for microcolonies incubated at neutral pH, where the log reduction is 0.78 CFU/ml (*P* < 0.05). To test for interferences of the hydrogel to bacterial acidic stress, planktonic populations cultivated in TSB were encased in agarose just before the survival test. Log reduction of these control planktonic populations suspended in agarose matrices presented significant and similar sensitivity than in planktonic TSB culture (*P* > 0.05), indicating no buffering effect of agarose to stomachal acidic stress (Fig. 5a).

## Discussion

The behaviour of microbial population in laboratory liquid growth media can strongly deviate from what is observed in real solid food matrices^18,30–34^. The environmental heterogeneity of structured media is listed as one of the four main causes of cellular variation, among genetic variation, ageing and stochasticity of gene expression^35^. As such, it can trigger a large diversity of phenotypic cell expression in the same biotope, promoting the cohabitation of cells with different spectra of behaviours, such as stress response or virulence^36,37^. Structured foods are not an optimal medium from an exploration perspective as the opacity of numerous food matrices prevents live imaging and microscopic approaches. To overcome these limitations, several studies take advantage of synthetic hydrogel systems to simplify and control the parameters of growth of embedded bacteria. Here, the agarose used to prepare the hydrogels was low melting point agarose (LMPA) which allowed to disperse the cells without thermal stress, with tuneable textures and media compositions able to mimic the texture of various food environments such as minced meat or soft cheese^29,38,39^. The experiments presented in this work show that the morphology of microcolonies in the media complemented with HCl is different from the neutral pH control, in particular bacteria are shedding from the periphery of the microcolonies. This effect can be explained by a combined action of relaxed gel structures due to low pH and the higher motility of *E. coli* O157:H7 when acidic conditions are encountered^29,40^. In fact, when examining individual cells of *E. coli* O157:H7 CM454 grown in 0.5% LPMA matrix at pH 5, we determined the mean speed (Smean) to be 0.97 µm/s and the maximum speed (Smax) to be 2.41 µm/s. Strikingly, no motility was observed at pH 7^29^.

In this study, we observed a clear overexpression of *gadB* for a subpopulation of cells localised at the periphery of microcolonies formed in acidic hydrogels. This gene is expressed at levels 2-3 times higher than in neutral conditions, which is in accordance with results obtained in planktonic conditions (Supplementary Fig. 2). We confirmed that this spatialisation was neither associated with dead cells (Supplementary Fig. 6), nor a limitation of oxygen for GFP maturation in the centre of the microcolony (Supplementary Fig. 3). The use of a single plasmid bearing both the genes for the constitutive and induced fluorescence means that, at the image analysis step, we prevented bias due to differences in plasmid copy numbers or differences in coloration from a mix of dye and genetic reporters^41^. The use of two lasers with different properties of penetration could lead to a bias in the *Z-*axis, but three-dimensional analysis of all the cells in a microcolony reduces the bias as any offset at the bottom of the agglomerate is compensated by an opposite offset at the top.

The combination of CSLM and a dual fluorescent reporter with a genetic amplification system has enabled the discovery of distinct spatial gene expression patterns in single cells within a microcolony. This finding emphasises that conventional RT-qPCR is inadequate for estimating gene expression levels in such scenarios, as it provides an average expression level across the entire population, concealing the heterogeneity of gene expression at the individual cell level and lacking spatial information. By employing fluorescent gene reporters in conjunction with microscopic observations, we can effectively capture gene expression heterogeneity and spatial distribution, making it a highly relevant approach. Spatial patterns of genetic expression were previously reported for other genes in other bacterial species in surface biofilms either on solid or liquid, such as localised expression of *E. coli* sigma factors and type 1 pili, as well as *Pseudomonas aeruginosa* β-lactamase in biofilms^42–44^. To our knowledge, such patterns of gene expression were never reported in hydrogels, food-like or food matrices. Then, we explored the consequences of growing populations in a structured media in regards of survival to an exposition to low pH media. Results showed that, regardless of the initial pH, populations incubated in a gellified media have a better tolerance to acid stress than those grown in liquid broths, where no surviving cells were detected. This underlines limitations in modelling food-borne pathogens behaviour in food from data obtained in liquid conditions, as previously shown in other studies^18,30,31,45^.

It has been suggested from other studies that the components of the matrix could have a buffer effect that protects embedded cells by preventing the drop in pH. Our tests show that planktonic populations dispersed in hydrogel did not present the same survival fitness that those cultivated as microcolonies in the same hydrogel. The hypothesis of a buffer effect due to hydrogel interference was tested for bacteria incubated in hydrogel but it was not supported statistically. This is reinforced by another study in a gellified dairy matrix, where food related bacteria were dispersed without incubation in the hydrogel before application of the acidic stress. It was reported that no protective effect existed compared to the same conditions in liquid media^46^.

A parameter that could explain this difference of survival between the populations incubated or not in the hydrogels would be the spatial organisation of cells. The hydrogels showed evidence of deliquescence such as unravelling of filaments and loss of stiffness but maintained enough structural integrity to ensure the microcolonies did not disperse. The ability of spatially organised populations of bacterial cells to better survive acid stress was described for pathogenic bacteria but also for auxiliary microbiota and probiotics, such as *Lactobacillus* strains^47,48^. The bacteria could secrete extracellular polymeric substances (EPS) when grown in communities as described in biofilms^49,50^. For *E. coli* O157:H7, tolerance of the bacteria to low pH could further involve the DNA binding protein Dps, which is known to enhance survivability when local nutrients are exhausted^51,52^.

From this study in a hydrogel matrix, *gadB* appeared to be more expressed at the periphery of the *E. coli* O157:H7 microcolonies in acidic conditions. This correlated with an increased tolerance to the type of acid stress that can be encountered by bacterial cells after ingestion of food. Experiments of co-cultures of *E. coli* O157:H7 and *L. lactis* demonstrate that the pattern of *gadB* expression could naturally occur in food matrix through a progressive accumulation and diffusion of lactic acid in the media, such as in cheese or meat products. The co-occurrence of lactic acid bacteria in foodstuff, which are generally considered probiotics, can significantly influence the physiology of foodborne pathogens, potentially leading to unforeseen consequences for food safety and risk assessment. These findings have significant implications for risk assessment and public health as they highlight inherent differences in bacterial physiology and virulence exist between liquid and solid food products. The contrasting characteristics observed underscore the need to consider the distinct challenges posed by these food types, thereby emphasising the importance of tailored risk mitigation strategies. In this case, the tolerance to acidity could mean an increase in the bacterial load that can survive in the digestive system as well as a phenotype more likely to colonise the gut lining. In order to alleviate public health issues, differences in bacterial behaviour in planktonic conditions versus microcolonies could be considered when integrating phenotypic heterogeneity in risk assessment. Modelling pathogens growth and survival should take in account the gelled environments where the spatialisation of genetic expression and its resulting populational effects could deeply affect foodborne pathogens behaviour and virulence after ingestion.

## Methods

### Bacterial strains and culture conditions

The *E. coli* O157:H7 CM454^53,54^ is the wild-type strain used in this study. From cryotubes stored at −80 °C, the bacterial strains of *E. coli* (see Genetic construction) were plated on Petri dishes with TSA (Tryptone Soya Agar, Oxoid, USA) and incubated overnight at 37 °C. One bacterial colony was picked up and inoculated in TSB (Oxoid, England) before overnight incubation at 37 °C under orbital shaking (200 rpm). When required, growth media were supplemented with chloramphenicol (Cm 25 μg/ml; EUROMEDEX, China). The strain *Lactococcus lactis* ssp. *cremoris* (AERIAL strain no. 2124; AERIAL Technological Resource Centre, Illkirch, France) was incubated in M17 (BD Difco) supplemented with 0.5% glucose.

### Dual fluorescent reporter system to monitor gene expression

To monitor the gene expression, we genetically engineered a dual fluorescent reporter system inspired from genetic amplification systems based on T7 polymerase (*T7pol*)^55,56^, where a *T7pol::Cm*^*R*^ cassette is inserted afterward the gene of interest (Supplementary Fig. 1). In the present case, insertion was performed downstream of the gene *gadB* in *E. coli* O157:H7 CM454 using the Datsenko–Wanner recombination technique^57^. For subsequent homologous recombination, regions of identity were added at the ends of the cassette using the forward primer gadBt7_FW 5’-CCGAAACTGCAGGGTATTGCCCAACAGAACAGCTTTAAACATACCTGATAACA GGAGGTAAATAATGCACACGATTAACATCGC3-’, and reverse primer gadBt7_RV 5’-AAATTGTCCCGAAACGGGTTCGTTTCGGACACCGTTACCGTTAAACATGGAGTTCTGAGGTCATTACTG-3’. The correct insertion of *T7pol::Cm*^*R*^ in the construct was verified by PCR using the forward primer 5’-GGAAGACTACAAAGCCTCCC-3’ and reverse primer 5’-TATTCCTGTCGGAACCGCAC-3’, for sequencing (Eurofins Genomics, Germany) (Supplementary Table 1). Based on the sequence of the pHL40 plasmid^55^, a new plasmid was synthetised (GeneArt, ThermoFisher Scientific, Germany) bearing the P_*T7pol*_*::GFPmut3::*T_*T7pol*_ as a green fluorescent protein (GFP) reporter but modified by insertion of P_*BBa_J23119*_*::mCherry2::*T_*BBa_B0062*_ (iGEM parts) for constitutive expression of the red fluorescent protein (RFP) mCherry2. This new plasmid, called pHL60, was transformed into competent *gadB::T7pol::Cm*^*R*^ bacterial cells. This system is an indirect reporter of *gadB* expression as the polycistronic expression of the T7 polymerase allows an amplified production of GFP (GFPmut3) from pHL60 and normalisation of the level of expression respective to the constitutive expression of the RFP (mCherry2) from the same plasmid, to minimise variations of the fluorescence associated with variations in the number of plasmids from one cell to another. To validate the genetic construction, the reporting planktonic expression of *gadB* was tested at six pH values, from 4.5 to 7.0, using a microplate reader (Synergy H1, Biotek) (Supplementary Fig. 2).

### Transparent hydrogels for fluorescent imaging

As previously described^29^, the hydrogels were obtained by mixing TSB with 0.50 % agarose, using low melting point agarose (LMPA) (UltraPure Agarose, Invitrogen, USA). After boiling, the liquid LMPA (pH 7) was cooled down to 40 °C to prevent thermal stress before the bacterial inoculum of *E. coli* O157:H7 CM454 was added to obtain 10^4^ CFU/ml, unless indicated otherwise. When necessary, the medium was adjusted to acidic pH 5 with HCl. After homogenising and gentle stirring to avoid bubble formation, 100 µl of the inoculated LMPA matrix was immediately distributed in each well of a 96-well microtiter plate of microscopic grade (µClear, Greiner Bio-One, France). The microtiter plates were then incubated at 20°C and observed under confocal laser scanning microscope (CLSM) after 96 h of incubation, unless indicated otherwise.

For in-gel co-culture experiments, *E. coli* O157:H7 CM454 was co-inoculated in 5 ml of neutral LMPA matrix (pH 7) as above at 10^3^, 10^4^ or 10^5^ CFU/ml, with *L. lactis* inoculated at 10^3^ CFU/ml. After homogenisation, 100 µl of the mixture was immediately deposited in wells of a 96-well microtiter plate and then incubated at 20 °C prior to CLSM observations at different time point as indicated.

### Confocal laser scanning microscope (CLSM) and image analysis

All microscopic observations were performed with a Leica HCS-SP8 CLSM at the INRAE MIMA2 imaging platform (https://www6.jouy.inrae.fr/mima2). The GFP (GFPmut3; *λ*_ex_500 nm; *λ*_em_513 nm) and RFP (mCherry2; *λ*_ex_589 nm; *λ*_em_610 nm) were excited, respectively, with laser bands 488 nm and 561 nm. Emitted fluorescence by GFP and RFP were sequentially collected on two independent hybrid detectors in the range 500–550 nm and 590–640 nm, respectively. For Live/dead exploration, SYTO9 (*λ*_ex_485 nm; *λ*_em_501 nm) and IP (*λ*_ex_535 nm; *λ*_em_617 nm) were excited respectively with laser bands 488 nm and 561 nm. Observations were carried out with a water immersion ×63 objective lens (numerical aperture of 1.20) for 184 µm × 184 µm fields. Bidirectional acquisition speed of 600 Hz allows a frame rate of 2.3 images per second. For 3D stack analysis, a 1 µm step between *z* levels was used. For each condition, a minimum of 60 stacks were acquired in over a dozen independent wells. Microscopic images were treated on IMARIS v9.64 (Bitplane, Switzerland) to generate sections and projections. The binarised volume of independent microcolonies was used to determine the mean radius of microcolonies in each condition. The red constitutive signal from fluorescent bacterial cells was used to define a sphere from which the microcolony radius was determined.

Kymograms reporting the spatial analysis of *gadB* expression in microcolonies were performed using BiofilmQ v0.2.2^58^. BiofilmQ image segmentation was performed with a threshold value set at 0.1 with cubes of 1.8 µm (vox of 10). The absence of radial fluorescence gradients in microcolonies of *E. coli* O157:H7 constitutively expressing GFP was verified prior experiments with *gadB* expression (Supplementary Fig. 3). A time-course microscopic analysis allows the observation of *gadB* expression spatialisation in acidic hydrogels as early as microcolonies become visible under the microscope, ~48 h after inoculation (data not shown).

### Acidic digestion challenge

To test the ability of *E. coli* O157:H7 population to survive the strong acidic stress during the stomachal passage, bacteria grown (72 h, 20 °C, pH 7 or 5) (i) as planktonic cells in TSB, (ii) as planktonic cells in TSB and then encased in LMPA matrix (TSB-Agarose), or (iii) as microcolonies in LMPA matrix (Agarose) were transferred (3 ml in 27 ml) in a saline solution of NaCl 9 g/l either neutral pH 7(control groups) or acidic pH 2 (adjusted with 5 M HCl). The cups were then incubated at 37 °C for 4 h under a 90-rpm shaking to simulate digestion. All media were then homogenised to disperse bacteria (IKA Ultra-Turrax T25; Janke Kunkel) and the resulting suspensions were immediately plated on TSA for enumeration and determination of the log reduction in CFU/ml before and after acidic treatment.

### Statistics

Graphics and ANOVA (analysis of variance) were performed with Prism 9 (GraphPad; CA, USA). Differences were considered significant when *P* < 0.05 with *P* being the critical probability associated with the Fisher test.

### Reporting summary

Further information on research design is available in the Nature Research Reporting Summary linked to this article.

### Supplementary information

Supplementary material

reporting summary

## Data Availability

Data that support the findings of this study are available within the article or its Supplementary Materials. Additional data can be made available upon request to the corresponding authors.
