# Supplementary material for: Spatially localised expression of the glutamate decarboxylase gadB in Escherichia coli O157:H7 microcolonies in hydrogel matrices
Source: NPJ Sci Food. 2023 Oct 14;7:55. doi: 10.1038/s41538-023-00229-8 (PMC10576782; doi:10.1038/s41538-023-00229-8)
Supplement: Supplementary file 1 — Supplementary material [file 41538_2023_229_MOESM1_ESM.pdf]

SUPPLEMENTARY MATERIALS

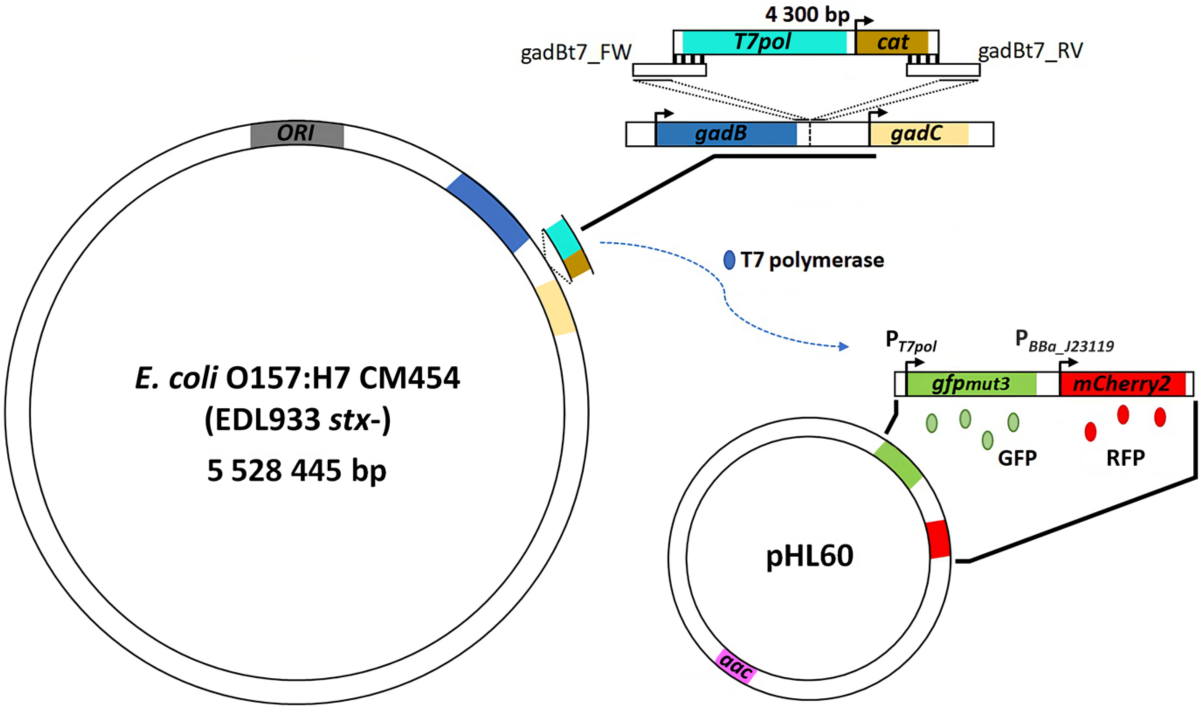

Supplementary Figure 1: Genetic strategy to monitor gene expression in *E. coli* O157:H7 using a dual fluorescent reporter with genetic amplification system. The picture depicts the chromosome of *E. coli* O157:H7 CM454 with a zoom in the region of insertion of the *T7pol::cat* cassette to indicate the different CDS locally present. The insertion site is located between the gene of interest *gadB* (in blue) and *gadC* (in yellow). The cassette bearing the gene encoding the T7 polymerase (*T7pol*) (in turquoise) and chloramphenicol resistance gene (in brown, Cm^R), i.e. *cat* (chloramphenicol O-acetyltransferase), was elongated by PCR with two primers (gadBt7_FW and gadBt7_RV) design for homologous recombination at the insertion site. Of note, *gabB* and *T7pol* form a newly synthetic polycistron under the control of the native *gadB* promoter. Besides, the low copy plasmid pHL60 bearing the fluorophore encoding genes is represented. It contains the constitutively expressed *mCherry2* (in red) and *gfpmut3* (in green) under the control of the *T7pol* promoter (in purple), as well as a gentamicin resistance gene (in pink; Gm^R), i.e. *aac* (aminoglycoside 3-N-acetyltransferase, AAC(3)-I family aminoglycoside), for selection. From a same plasmid, the dual fluorescent reporter system here allows an amplified and indirect reporter of *gadB* expression thanks to the polycistronic expression of the T7 polymerase (which induces expression of GFP), whereas the constitutive expression of the red fluorescence protein (RFP) mCherry2 allows to normalise the expression level of GFP (*e.g.* in case of potential variations in the number of plasmids from one bacterial cell to another).

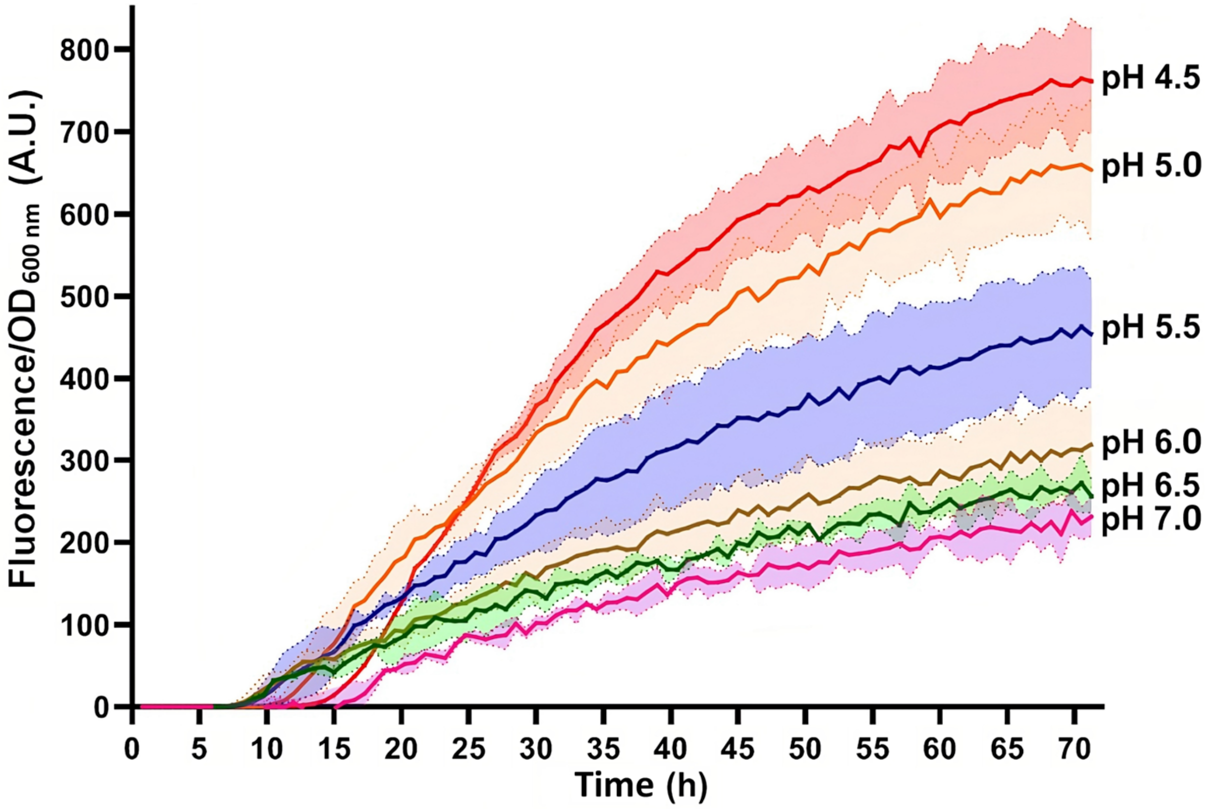

Supplementary Figure 2: Relative expression of *gadB* in a population of *E. coli* O157:H7 cells as a function of time and under different acidity levels. The fluorescence of planktonic populations of *E. coli* O157:H7 *gadB::GFPmut3* are represented in function of the $OD_{600\text{ nm}}$. For each curve representing the mean signal over time, the standard deviation of values is shown as an area of lighter color. These curves show that detected fluorescence becomes incrementally brighter with decreasing pH. The increase in expressed fluorescence is not linear, with greater leaps of intensity below pH 6.0. Points over time show that fluorescence intensity at pH 4.5 can be 3-4 times higher than reported values for the control at pH 7.0. The lag time before the green fluorescence is detected is high at pH 7.0 (15 h) compared to pH 5.5 where it starts after eight hours. In media pH 5.0 and pH 4.5, the lag time becomes progressively longer (12 then 14 h).

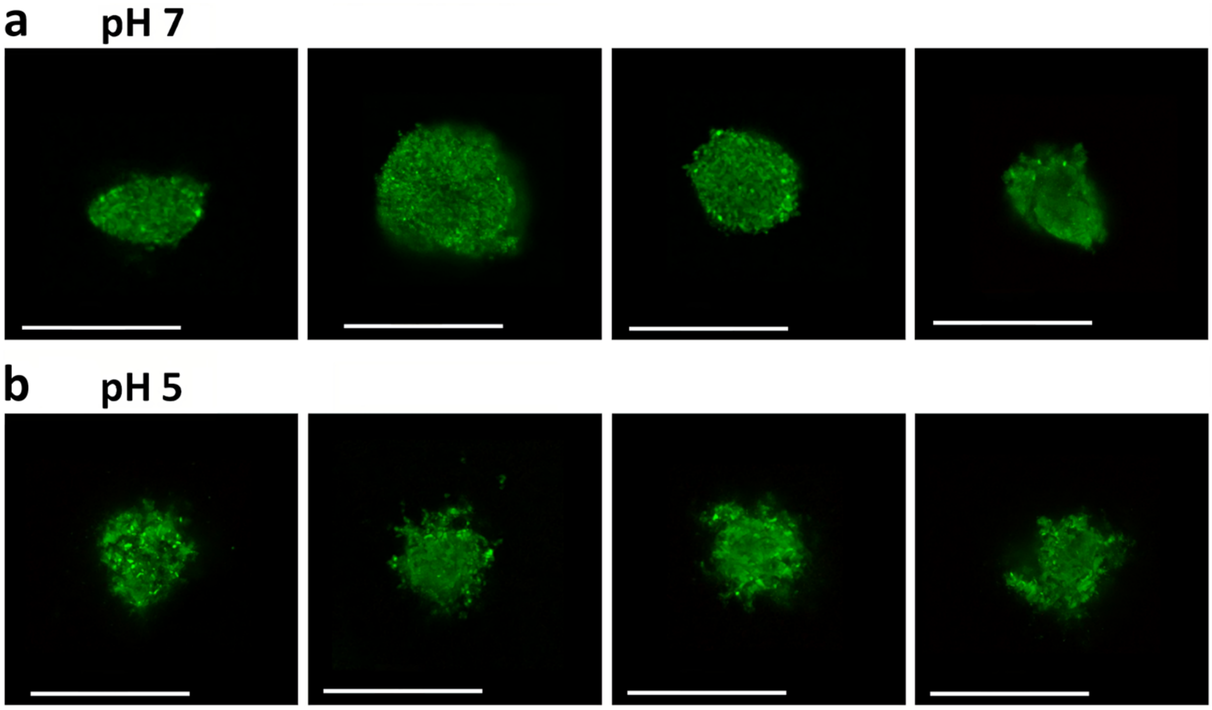

Supplementary Figure 3: Qualitative presentation of the constitutive GFP spatial expression in microcolonies of *E. coli* O157:H7 in neutral (pH 7) or acidic (pH 5) hydrogels. Representative microscopic observations were performed in neutral LMPA matrix (pH 7, a) and in acid LMPA matrix (pH 5, b). Length of the scale bars represents 50 µm. Each image is a 5 µm thick slice of a stack. Control experiments performed with a constitutive expression of GFP did not show any spatialisation of the expression. Time-course microscopic analysis allowed the observation of *gadB* expression in acidic hydrogels as early as microcolonies become visible under the microscope, ~48 h after inoculation (data not shown).

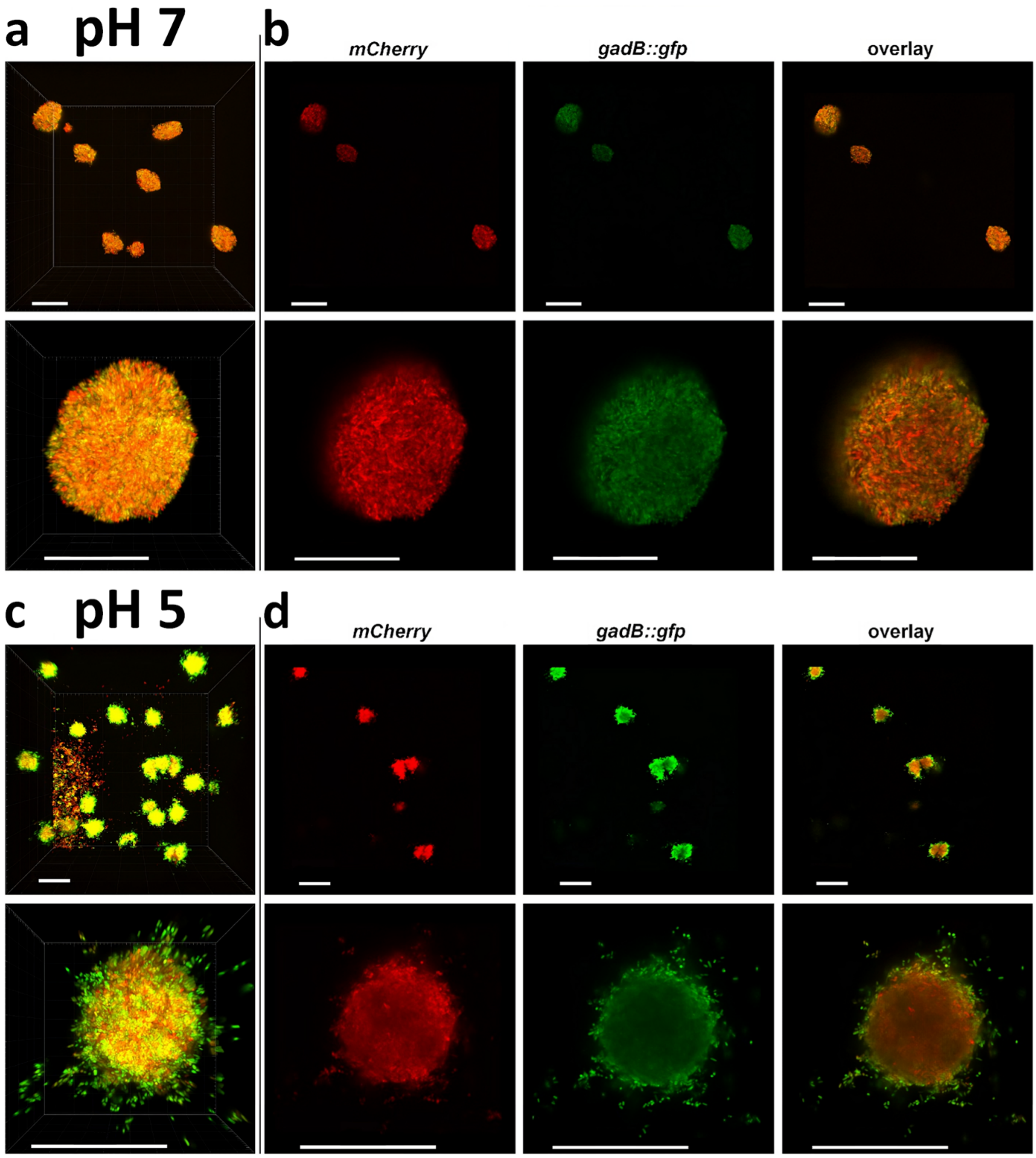

Supplementary Figure 4: Qualitative presentation of *gadB* spatial expression in microcolonies of *E. coli* O157:H7 in neutral (pH 7) or acidic (pH 5) hydrogels. Representative microscopic observations of the expression of *gadB* in neutral LMPA matrix (pH 7, a and b) and in acid LMPA matrix (pH 5, c and d). For both groups, the 2 successive rows use a 40X then a 63X objective to present a collection of microcolonies and a close observation of a single representative microcolony. Length of the scale bars represents 50 µm. Panels a and c correspond to three-dimensional representations. Panels b and d correspond 5 µm slices showing both fluorescent channels and the overlay. The first row of each condition was taken with a 40X air objective (numerical aperture = 0.85) to explore a 290 µm x 290 µm fields.

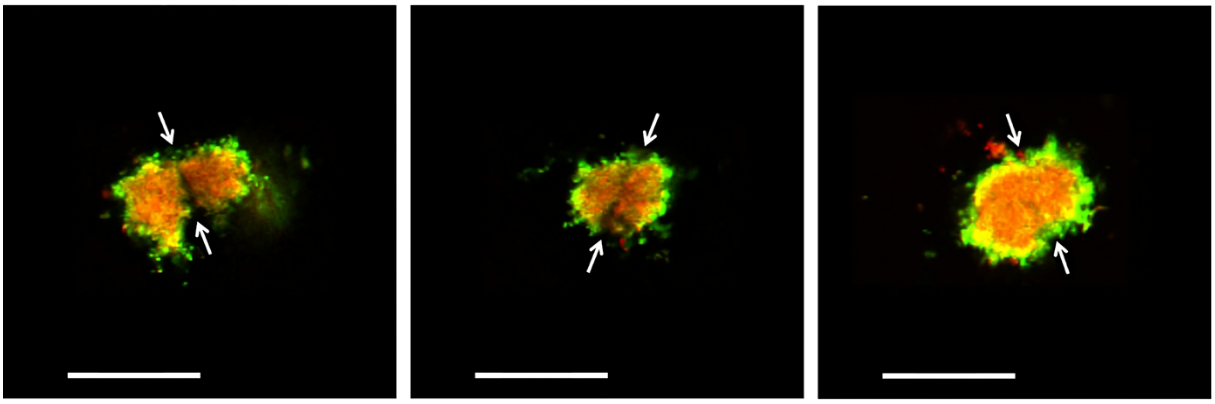

Supplementary Figure 5: The case of joint microcolonies of *E. coli* O157:H7. Representative examples where two microcolonies of *E. coli* O157:H7 are touching or merging in hydrogels at pH 5. White arrows indicate the separation between each microcolonies. Length of the scale bars represents 50 µm.

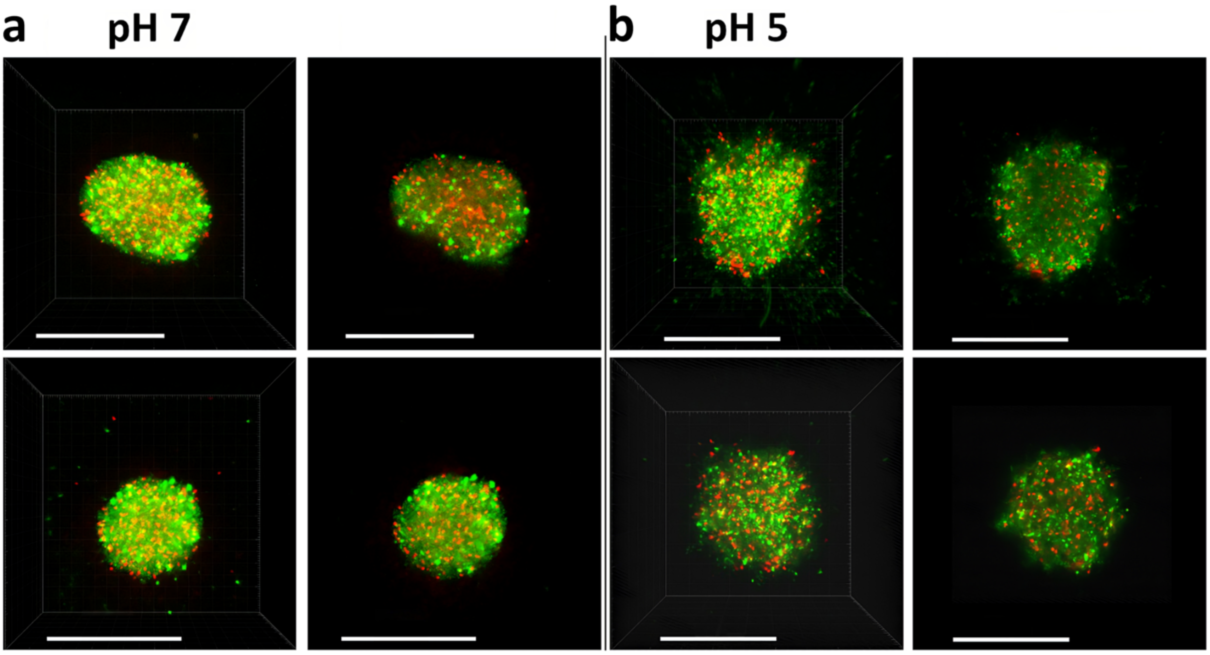

Supplementary Figure 6: Live/Dead staining in microcolonies of *E. coli* O157:H7. Bacterial cells in two representative microcolonies grown in hydrogels at neutral (pH 7, a) or acidic (pH 5, b) conditions were labelled with the cell impermeant propidium iodine (red, dead cells) and the cell permeant SYTO9 (green, all cells). In each series of two images (from left to right), the first corresponds to a three-dimensional representation and the second to a 5 µm slice. Length of the scale bars represents 50 µm. The spatial repartition of dead cells in microcolonies as shown by live/dead fluorescent staining indicated a random distribution of the red dead cells, with no preferential localisation in the microcolonies associated with cells expressing *gadB*.

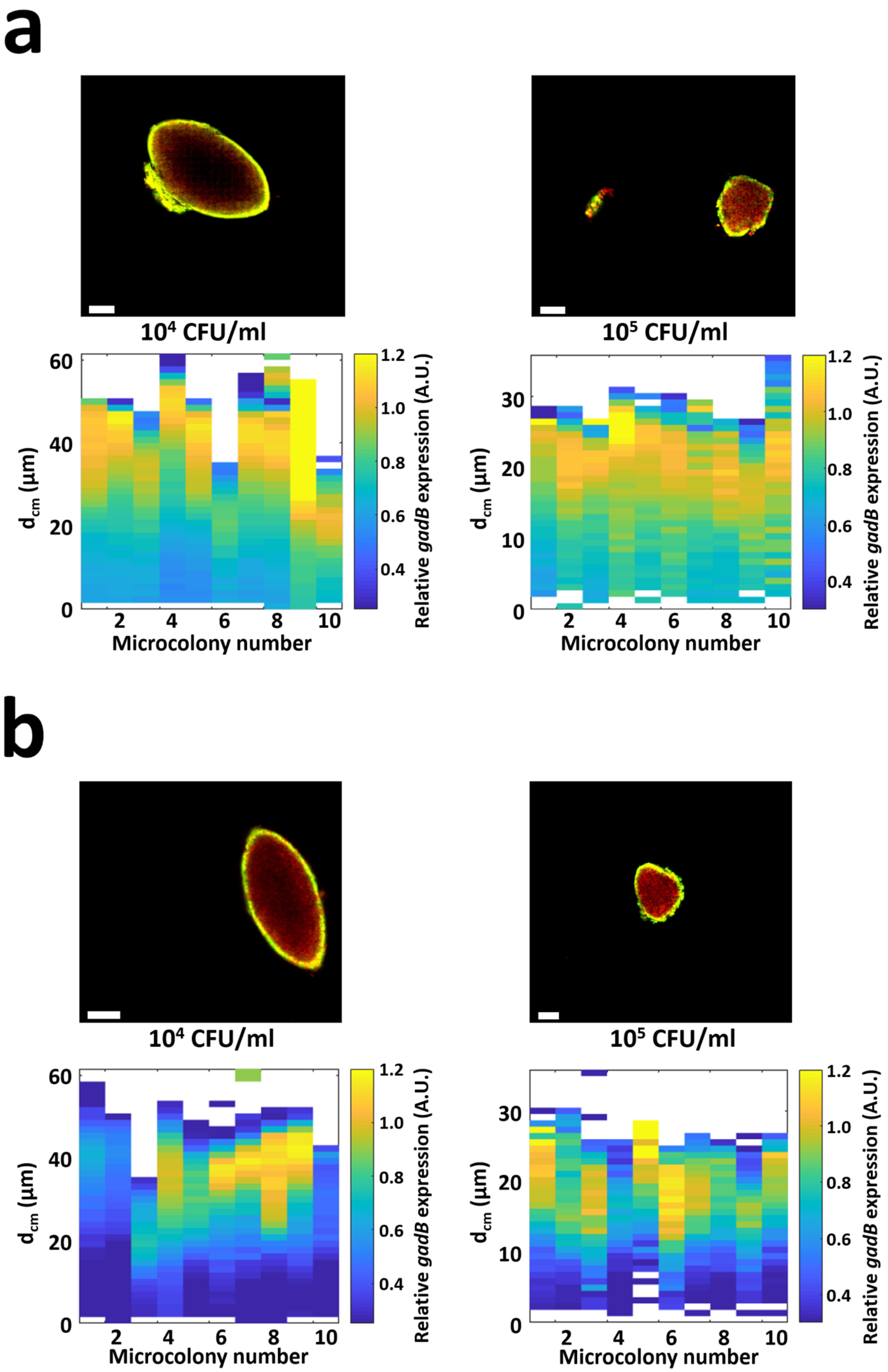
a
10^4 CFU/ml
10^5 CFU/ml
d_cm (μm)
Microcolony number
Relative gadB expression (A.U.)
60
40
20
0
30
10
2
4
6
8
10
1.2
1.0
0.8
0.6
0.4
b
10^4 CFU/ml
10^5 CFU/ml
d_cm (μm)
Microcolony number
Relative gadB expression (A.U.)

Supplementary Figure 7: Spatial patterns and kymograms of *gadB* expression in *E. coli* O157:H7 microcolonies in the presence of *L. lactis*, cultivated in neutral (pH 7) hydrogels before and after acidic exposure (HCl pH 2). (a) Representative microscopic observations and kymograms of the relative *gadB* expression before acidic exposure. (b) Representative microscopic observations and kymograms of the relative *gadB* expression after acidic exposure. Pictures illustrates representative microscopic observations in the respective conditions. Length of the scale bars represents 50 µm. The relative *gadB* expression is provided as the ratio of green fluorescence intensity over red fluorescent intensity (GFP reporter/RFP constitutive) as a function of the distance from the center of the microcolony (dCM) from in-gel microcolonies of *E. coli* O157:H7 co-inoculated (at 10^4 or 10^5 CFU/ml) with *L. lactis* (inoculated at 10^3 CFU/ml). Data results from 10 independent microcolonies in the respective conditions.

Supplementary Table 1: Insertion of *T7pol::Cm*^{R} in the *gadB-gadC* intergenic region of *E. coli* O157:H7 CM454. CDS of the targeted DNA region, expected genetic construct after chromosomic insertion, and sequencing results demonstrating the correct insertion of the *T7pol::Cm*^{R} reporter cassette in the intergenic *gadB-gadC* region.

Targeted DNA region

GenBank ID: CP008957.1 DNA location: 2002495-2005586 (*E. coli* O157:H7 EDL933)

CDS: *gadB gadC*

Nucleotide sequence:

ATGGATAAGAAGCAAGTAACGGATTTAAGGTCGGAACTACTCGATTCACGTTTTGGTGCGAAGTCTATTTCCACTATCGCAGAATCAAAACGTTTT
CCGCTGCACGAAATGCGCGACGATGTCGCATTTCAGATTATCAATGACGAATTATATCTTGATGGCAACGCTCGTCAGAACCTGGCCACTTTCTGC
CAGACCTGGGACGACGAAAACGTCCACAAGTTGATGGATTTATCCATTAACAAAAACTGGATCGACAAAGAAGAATATCCGCAATCTGCAGCCATC
GACCTGCGTTGCGTAAATATGGTTGCCGATCTGTGGCATGCGCCTGCGCCGAAAAATGGTCAGGCCGTTGGCACCAACACCATTGGTTCTTCCGAG
GCCTGTATGCTCGGCGGGATGGCGATGAAATGGCGTTGGCGCAAGCGTATGGAAGCTGCAGGCAAACCAACGGATAAACCAAACCTGGTGTGCGGC
CCGGTGCAAATCTGCTGGCATAAATTCGCCCGCTACTGGGATGTGGAGCTGCGTGAGATCCCTATGCGCCCCGGTCAGTTGTTTATGGATCCGAAA
CGCATGATTGAAGCCTGCGACGAAAATACCATCGGCGTGGTGCCGACTTTCGGCGTGACCTACACCGGTAACTATGAGTTCCCGCAACCGCTGCAC
GATGCGCTGGATAAATTCCAGGCCGACACCGGTATCGACATCGACATGCACATCGACGCCGCCAGTGGTGGCTTCCTGGCACCGTTCGTCGCCCCG
GATATCGTCTGGGACTTCCGCCTGCCGCGTGTGAAATCGATCAGTGCTTCAGGCCACAAATTCGGTCTGGCTCCGCTGGGCTGCGGCTGGGTTATC
TGGCGTGATGAAGAAGCGCTGCCGCAGGAACTGGTGTTCAACGTTGACTACCTCGGTGGTCAGATTGGTACTTTCGCCATCAACTTCTCCCGCCCG
GCGGGTCAGGTAATTGCACAGTACTATGAATTCCTGCGCCTCGGTCGTGAAGGCTATACCAAAGTACAGAACGCCTCTTACCAGGTTGCCGCTTAT
CTGGCGGATGAAATCGCCAAACTGGGGCCGTATGAGTTCATCTGTACCGGTCGCCCGGACGAAGGCATTCCGGCGGTTTGCTTCAAACTGAAAGAT
GGTGAAGATCCGGGATACACCCTGTATGACCTCTCTGAACGTCTGCGTCTGCGCGGCTGGCAGGTTCCGGCCTTCACTCTCGGCGGTGAAGCCACC
GACATCGTGGTGATGCGCATTATGTGTCGTCGCGGCTTCGAAATGGACTTTGCTGAACTGTTGCTGGAAGACTACAAAGCCTCCCTGAAATATCTC
*AGTGATCACCCGAAACTGCAGGGTATTGCCCAACAGAACAGCTTTAAACATACCTGA*TAACGTTTAACGGTAACGGTGTCCCGAAACGAACCCGTT
TCGGGACAATTTCCAAAGTCTGTTCACTGGCATTAGCAACGGAAAATATTGTTCTGAATACGCTTCAGAACAAAACAGGTGCGGTTCCGACAGGAA
TACCGTTTTAGGGGGATAAT*ATGGCTACATTAGTACAGACAGGTAAAGCTAAGCAGCTCACATTACTCGGCTTTTTTGCCATAACGGCATCGATGG*
TAATGGCTGTTTATGAATACCCTACCTTCGCAACATCGGGCTTTTCATTAGTCTTCTTCCTGCTATTAGGCGGGATTTTATGGTTTATTCCCGTGG
GACTTTGTGCTGCGGAAATGGCCACCGTCGACGGCTGGGAAGAAGGTGGTGTCTTCGCCTGGGTATCAAATACTCTGGGGCCGAGATGGGGATTTG
CAGCGATCTCATTTGGCTATCTGCAAATCGCCATTGGTTTTATTCCGATGCTCTATTTCGTGTTAGGGGCACTCTCCTACATCCTGAAATGGCCAG
CGCTGAATGAAGACCCCATTACCAAAACTATTGCAGCACTCATCATTCTTTGGGCGCTGGCATTAACGCAGTTTGGTGGCACGAAATACACGGCGC
GAATTGCTAAAGTTGGCTTCTTCGCCGGTATCCTGTTACCTGCATTTATTTTGATTGCATTAGCGGCTATTTATCTGCACTCCGGTGCCCCCGTTG
CTATCGAAATGGATTCGAAGACCTTCTTCCCTGACTTCTCTAAAGTGGGCACCCTGGTTGTATTTGTTGCCTTCATTTTGAGTTATATGGGCGTAG
AAGCTTCCGCAACCCACGTCAATGAAATGAGTAACCCAGGGCGCGACTATCCACTGGCTATGTTACTGCTGATGGTGGCGGCAATCTGCTTAAGCT
CTGTTGGCGGTTTGTCTATTGCGATGGTCATTCCGGGTAATGAAATCAACCTCTCCGCAGGGGTAATGCAAACCTTTACCGTTCTGATGTCCCATG
TGGCACCGGAAATTGAGTGGACGGTTCGCGTGATCTCCGCACTGCTGTTGCTGGGTGTTCTGGCGGAAATCGCCTCCTGGATTGTTGGTCCTTCTC
GCGGGATGTATGTCACAGCGCAGAAAAACCTGCTGCCAGCGGCATTCGCTAAAATGAACAAAAATGGCGTACCGGTAACGCTGGTCATTTCGCAGC
TGGTGATTACTTCTATCGCGTTGATCATCCTCACCAATACCGGTGGCGGTAACAACATGTCCTTCCTGATCGCACTGGCGCTGACGGTGGTGATTT
ATCTGTGTGCTTATTTCATGCTGTTTATTGGCTACATTGTGTTGGTTCTTAAACATCCTGACTTAAAACGCACATTTAATATCCCTGGTGGTAAAG
GGGTGAAACTGGTCGTGGCAATTGTCGGTCTGCTGACTTCAATTATGGCGTTTATTGTTTCCTTCCTGCCGCCGGATAACATCCAGGGTGACTCTA
CCGATATGTATGTTGAATTACTGGTCGTTAGTTTCCTGGTGGTACTTGCCCTGCCCTTTATTCTCTATGCTGTTCATGATCGTAAAGGCAAAGCGA
ATACCGGCGTCACTCTGGAGCCAATCAACAGTCAGAACGCACCAAAAGGTCACTTCTTCCTGCACCCGCGTGCACGTTCACCACACTATATTGTGA
TGAATGACAAGAAACACTAA

CDS: *gadB*
Locus tag: EDL933_2147 Locus-tag correspondence: Z2215
GenBank ID: CP008957.1 DNA location: 2002495-2003895
Protein name: Glutamate decarboxylase EC number: 4.1.1.15 Protein ID: AIG68335.1

Nucleotide sequence:

ATGGATAAGAAGCAAGTAACGGATTTAAGGTCGGAACTACTCGATTCACGTTTTGGTGCGAAGTCTATTTCCACTATCGCAGAATCAAAACGTTTT
CCGCTGCACGAAATGCGCGACGATGTCGCATTTCAGATTATCAATGACGAATTATATCTTGATGGCAACGCTCGTCAGAACCTGGCCACTTTCTGC
CAGACCTGGGACGACGAAAACGTCCACAAGTTGATGGATTTATCCATTAACAAAAACTGGATCGACAAAGAAGAATATCCGCAATCTGCAGCCATC
GACCTGCGTTGCGTAAATATGGTTGCCGATCTGTGGCATGCGCCTGCGCCGAAAAATGGTCAGGCCGTTGGCACCAACACCATTGGTTCTTCCGAG
GCCTGTATGCTCGGCGGGATGGCGATGAAATGGCGTTGGCGCAAGCGTATGGAAGCTGCAGGCAAACCAACGGATAAACCAAACCTGGTGTGCGGC
CCGGTGCAAATCTGCTGGCATAAATTCGCCCGCTACTGGGATGTGGAGCTGCGTGAGATCCCTATGCGCCCCGGTCAGTTGTTTATGGATCCGAAA
CGCATGATTGAAGCCTGCGACGAAAATACCATCGGCGTGGTGCCGACTTTCGGCGTGACCTACACCGGTAACTATGAGTTCCCGCAACCGCTGCAC
GATGCGCTGGATAAATTCCAGGCCGACACCGGTATCGACATCGACATGCACATCGACGCCGCCAGTGGTGGCTTCCTGGCACCGTTCGTCGCCCCG
GATATCGTCTGGGACTTCCGCCTGCCGCGTGTGAAATCGATCAGTGCTTCAGGCCACAAATTCGGTCTGGCTCCGCTGGGCTGCGGCTGGGTTATC
TGGCGTGATGAAGAAGCGCTGCCGCAGGAACTGGTGTTCAACGTTGACTACCTCGGTGGTCAGATTGGTACTTTCGCCATCAACTTCTCCCGCCCG
GCGGGTCAGGTAATTGCACAGTACTATGAATTCCTGCGCCTCGGTCGTGAAGGCTATACCAAAGTACAGAACGCCTCTTACCAGGTTGCCGCTTAT
CTGGCGGATGAAATCGCCAAACTGGGGCCGTATGAGTTCATCTGTACCGGTCGCCCGGACGAAGGCATTCCGGCGGTTTGCTTCAAACTGAAAGAT
GGTGAAGATCCGGGATACACCCTGTATGACCTCTCTGAACGTCTGCGTCTGCGCGGCTGGCAGGTTCCGGCCTTCACTCTCGGCGGTGAAGCCACC
GACATCGTGGTGATGCGCATTATGTGTCGTCGCGGCTTCGAAATGGACTTTGCTGAACTGTTGCTGGAAGACTACAAAGCCTCCCTGAAATATCTC
AGTGATCACCCGAAACTGCAGGGTATTGCCCAACAGAACAGCTTTAAACATACCTGA

Protein sequence:

MDKKQVTDLRSELLDSRFGAKSISTIAESKRFPLHEMRDDVAFQIINDELYLDGNARQNLATFCQTWDDENVHKLMDLSINKNWIDKEEYPQSAAI
DLRCVNMVADLWHAPAPKNGQAVGTNTIGSSEACMLGGMAMKWRWRKRMEAAGKPTDKPNLVCGPVQICWHKFARYWDVELREIPMRPGQLFMDPK
RMIEACDENTIGVVPTFGVTYTGNYEFPQPLHDALDKFQADTGIDIDMHIDAASGGFLAPFVAPDIVWDFRLPRVKSISASGHKFGLAPLGCGWVI
WRDEEALPQELVFNVDYLGGQIGTFAINFSRPAGQVIAQYYEFLRLGREGYTKVQNASYQVAAYLADEIAKLGPYEFICTGRPDEGIPAVCFKLKD
GEDPGYTLYDLSERLRLRGWQVPAFTLGGEATDIVVMRIMCRRGFEMDFAELLLEDYKASLKYLSDHPKLQGIAQQNSFKHT

CDS: *gadC*
Locus tag: EDL933_2148 Locus-tag correspondence: Z2216
GenBank ID: CP008957.1 DNA location: 2004051-2005586
Protein name: glutamate/gamma-aminobutyrate antiporter Protein ID: AIG68336.1

Nucleotide sequence:

ATGGCTACATTAGTACAGACAGGTAAAGCTAAGCAGCTCACATTACTCGGCTTTTTTGCCATAACGGCATCGATGGTAATGGCTGTTTATGAATAC
CCTACCTTCGCAACATCGGGCTTTTCATTAGTCTTCTTCCTGCTATTAGGCGGGATTTTATGGTTTATTCCCGTGGGACTTTGTGCTGCGGAAATG
GCCACCGTCGACGGCTGGGAAGAAGGTGGTGTCTTCGCCTGGGTATCAAATACTCTGGGGCCGAGATGGGGATTTGCAGCGATCTCATTTGGCTAT
CTGCAAATCGCCATTGGTTTTATTCCGATGCTCTATTTCGTGTTAGGGGCACTCTCCTACATCCTGAAATGGCCAGCGCTGAATGAAGACCCCATT
ACCAAAACTATTGCAGCACTCATCATTCTTTGGGCGCTGGCATTAACGCAGTTTGGTGGCACGAAATACACGGCGCGAATTGCTAAAGTTGGCTTC
TTCGCCGGTATCCTGTTACCTGCATTTATTTTGATTGCATTAGCGGCTATTTATCTGCACTCCGGTGCCCCCGTTGCTATCGAAATGGATTCGAAG
ACCTTCTTCCCTGACTTCTCTAAAGTGGGCACCCTGGTTGTATTTGTTGCCTTCATTTTGAGTTATATGGGCGTAGAAGCTTCCGCAACCCACGTC
AATGAAATGAGTAACCCAGGGCGCGACTATCCACTGGCTATGTTACTGCTGATGGTGGCGGCAATCTGCTTAAGCTCTGTTGGCGGTTTGTCTATT
GCGATGGTCATTCCGGGTAATGAAATCAACCTCTCCGCAGGGGTAATGCAAACCTTTACCGTTCTGATGTCCCATGTGGCACCGGAAATTGAGTGG
ACGGTTCGCGTGATCTCCGCACTGCTGTTGCTGGGTGTTCTGGCGGAAATCGCCTCCTGGATTGTTGGTCCTTCTCGCGGGATGTATGTCACAGCG
CAGAAAAACCTGCTGCCAGCGGCATTCGCTAAAATGAACAAAAATGGCGTACCGGTAACGCTGGTCATTTCGCAGCTGGTGATTACTTCTATCGCG
TTGATCATCCTCACCAATACCGGTGGCGGTAACAACATGTCCTTCCTGATCGCACTGGCGCTGACGGTGGTGATTTATCTGTGTGCTTATTTCATG
CTGTTTATTGGCTACATTGTGTTGGTTCTTAAACATCCTGACTTAAAACGCACATTTAATATCCCTGGTGGTAAAGGGGTGAAACTGGTCGTGGCA
ATTGTCGGTCTGCTGACTTCAATTATGGCGTTTATTGTTTCCTTCCTGCCGCCGGATAACATCCAGGGTGACTCTACCGATATGTATGTTGAATTA

CTGGTCGTTAGTTTCCTGGTGGTACTTGCCCTGCCCTTTATTCTCTATGCTGTTCATGATCGTAAAGGCAAAGCGAATACCGGCGTCACTCTGGAG
CCAATCAACAGTCAGAACGCACCAAAAGGTCACTTCTTCCTGCACCCGCGTGCACGTTCACCACACTATATTGTGATGAATGACAAGAAACACTAA

Protein sequence:

MATLVQTGKAKQLTLLGFFAITASMVMAVYEYPTFATSGFSLVFFLLLGGILWFIPVGLCAAEMATVDGWEEGGVFAWVSNTLGPRWGFAAISFGY
LQIAIGFIPMLYFVLGALSYILKWPALNEDPITKTIAALIILWALALTQFGGTKYTARIAKVGFFAGILLPAFILIALAAIYLHSGAPVAIEMDSK
TFFPDFSKVGTLVVFVAFILSYMGVEASATHVNEMSNPGRDYPLAMLLLMVAAICLSSVGGLSIAMVIPGNEINLSAGVMQTFTVLMSHVAPEIEW
TVRVISALLLLGVLAEIASWIVGPSRGMYVTAQKNLLPAAFAKMNKNGVPVTLVISQLVITSIALIILTNTGGGNNMSFLIALALTVVIYLCAYFM
LFIGYIVLVLKHPDLKRTFNIPGGKGVKLVVAIVGLLTSIMAFIVSFLPPDNIQGDSTDMYVELLVVSFLVVLALPFILYAVHDRKGKANTGVTLE
PINSQNAPKGHFFLHPRARSPHYIVMNDKKH

Primers:

gadBt7_FW: CCGAAACTGCAGGGTATTGCCCAACAGAACAGCTTTAAACATACCTGATAAC**AGGAGGTAAATAATGCACACGATTAACATCGC**
gadBt7_RV: AAATTGTCCCGAAACGGGTTCGTTTCGGACACCGTTACCGTTAAAC**ATGGAGTTCTGAGGTCATTACTG**
OutSeqGad_FW: GGAAGACTACAAAGCCTCCC
OutSeqGad_RV: TATTCCTGTCGGAACCGCAC

Expected genetic construct after chromosomic insertion

CDS: *gadB*
T7pol
cat

Primers: OutSeqGad FW
gadBt7 FW
gadBt7 RV
OutSeqGad RV

GGAAGACTACAAAGCCTCCCTGAAATATCTCAGTGATCACCCGAAACTGCAGGGTATTGCCCAACAGAACAGCTTTAAACATACCTGATAAC**AGGA**
GGTAAATA***ATGCACACGATTAACATCGC****TAAGAACGACTTCTCTGACATCGAACTGGCTGCTATCCCGTTCAACACTCTGGCTGACCATTACGGTG*
AGCGTTTAGCTCGCGAACAGTTGGCCCTTGAGCATGAGTCTTACGAGATGGGTGAAGCACGCTTCCGCAAGATGTTTGAGCGTCAACTTAAAGCTG
GTGAGGTTGCGGATAACGCTGCCGCCAAGCCTCTCATCACTACCCTACTCCCTAAGATGATTGCACGCATCAACGACTGGTTTGAGGAAGTGAAAG
CTAAGCGCGGCAAGCGCCCGACAGCCTTCCAGTTCCTGCAAGAAATCAAGCCGGAAGCCGTAGCGTACATCACCATTAAGACCACTCTGGCTTGCC
TAACCAGTGCTGACAATACAACCGTTCAGGCTGTAGCAAGCGCAATCGGTCGGGCCATTGAGGACGAGGCTCGCTTCGGTCGTATCCGTGACCTTG
AAGCTAAGCACTTCAAGAAAAACGTTGAGGAACAACTCAACAAGCGCGTAGGGCACGTCTACAAGAAAGCATTTATGCAAGTTGTCGAGGCTGACA
TGCTCTCTAAGGGTCTACTCGGTGGCGAGGCGTGGTCTTCGTGGCATAAGGAAGACTCTATTCATGTAGGAGTACGCTGCATCGAGATGCTCATTG
AGTCAACCGGAATGGTTAGCTTACACCGCCAAAATGCTGGCGTAGTAGGTCAAGACTCTGAGACTATCGAACTCGCACCTGAATACGCTGAGGCTA
TCGCAACCCGTGCAGGTGCGCTGGCTGGCATCTCTCCGATGTTCCAACCTTGCGTAGTTCCTCCTAAGCCGTGGACTGGCATTACTGGTGGTGGCT
ATTGGGCTAACGGTCGTCGTCCTCTGGCGCTGGTGCGTACTCACAGTAAGAAAGCACTGATGCGCTACGAAGACGTTTACATGCCTGAGGTGTACA
AAGCGATTAACATTGCGCAAAACACCGCATGGAAAATCAACAAGAAAGTCCTAGCGGTCGCCAACGTAATCACCAAGTGGAAGCATTGTCCGGTCG
AGGACATCCCTGCGATTGAGCGTGAAGAACTCCCGATGAAACCGGAAGACATCGACATGAATCCTGAGGCTCTCACCGCGTGGAAACGTGCTGCCG
CTGCTGTGTACCGCAAGGACAAGGCTCGCAAGTCTCGCCGTATCAGCCTTGAGTTCATGCTTGAGCAAGCCAATAAGTTTGCTAACCATAAGGCCA
TCTGGTTCCCTTACAACATGGACTGGCGCGGTCGTGTTTACGCTGTGTCAATGTTCAACCCGCAAGGTAACGATATGACCAAAGGACTGCTTACGC
TGGCGAAAGGTAAACCAATCGGTAAGGAAGGTTACTACTGGCTGAAAATCCACGGTGCAAACTGTGCGGGTGTCGATAAGGTTCCGTTCCCTGAGC
GCATCAAGTTCATTGAGGAAAACCACGAGAACATCATGGCTTGCGCTAAGTCTCCACTGGAGAACACTTGGTGGGCTGAGCAAGATTCTCCGTTCT
GCTTCCTTGCGTTCTGCTTTGAGTACGCTGGGGTACAGCACCACGGCCTGAGCTATAACTGCTCCCTTCCGCTGGCGTTTGACGGGTCTTGCTCTG
GCATCCAGCACTTCTCCGCGATGCTCCGAGATGAGGTAGGTGGTCGCGCGGTTAACTTGCTTCCTAGTGAAACCGTTCAGGACATCTACGGGATTG
TTGCTAAGAAAGTCAACGAGATTCTACAAGCAGACGCAATCAATGGGACCGATAACGAAGTAGTTACCGTGACCGATGAGAACACTGGTGAAATCT
CTGAGAAAGTCAAGCTGGGCACTAAGGCACTGGCTGGTCAATGGCTGGCTTACGGTGTTACTCGCAGTGTGACTAAGCGTTCAGTCATGACGCTGG
CTTACGGGTCCAAAGAGTTCGGCTTCCGTCAACAAGTGCTGGAAGATACCATTCAGCCAGCTATTGATTCCGGCAAGGGTCTGATGTTCACTCAGC
CGAATCAGGCTGCTGGATACATGGCTAAGCTGATTTGGGAATCTGTGAGCGTGACGGTGGTAGCTGCGGTTGAAGCAATGAACTGGCTTAAGTCTG
CTGCTAAGCTGCTGGCTGCTGAGGTCAAAGATAAGAAGACTGGAGAGATTCTTCGCAAGCGTTGCGCTGTGCATTGGGTAACTCCTGATGGTTTCC
CTGTGTGGCAGGAATACAAGAAGCCTATTCAGACGCGCTTGAACCTGATGTTCCTCGGTCAGTTCCGCTTACAGCCTACCATTAACACCAACAAAG
ATAGCGAGATTGATGCACACAAACAGGAGTCTGGTATCGCTCCTAACTTTGTACACAGCCAAGACGGTAGCCACCTTCGTAAGACTGTAGTGTGGG
CACACGAGAAGTACGGAATCGAATCTTTTGCACTGATTCACGACTCCTTCGGTACCATTCCGGCTGACGCTGCGAACCTGTTCAAAGCAGTGCGCG
AAACTATGGTTGACACATATGAGTCTTGTGATGTACTGGCTGATTTCTACGACCAGTTCGCTGACCAGTTGCACGAGTCTCAATTGGACAAAATGC
*CAGCACTTCCGGCTAAAGGTAACTTGAACCTCCGTGACATCTTAGAGTCGGACTTCGCGTTCGCGTAA*GGGCCCATATCTGGCGAAAATGAGACGT
TGATCGGCACGTAAGAGGTTCCAACTTTCACCATAATGAAATAAGATCACTACCGGGCGTATTTTTTGAGTTATCGAGATTTTCAGGAGCTAAGGA
AGCTAAA*ATGGAGAAAAAAATCACTGGATATACCACCGTTGATATATCCCAATGGCATCGTAAAGAACATTTTGAGGCATTTCAGTCAGTTGCTCA*
ATGTACCTATAACCAGACCGTTCAGCTGGATATTACGGCCTTTTTAAAGACCGTAAAGAAAAATAAGCACAAGTTTTATCCGGCCTTTATTCACAT
TCTTGCCCGCCTGATGAATGCTCATCCGGAATTCCGTATGGCAATGAAAGACGGTGAGCTGGTGATATGGGATAGTGTTCACCCTTGTTACACCGT
TTTCCATGAGCAAACTGAAACGTTTTCATCGCTCTGGAGTGAATACCACGACGATTTCCGGCAGTTTCTACACATATATTCGCAAGATGTGGCGTG

TTACGGTGAAAACCTGGCCTATTTCCCTAAAGGGTTTATTGAGAATATGTTTTTCGTCTCAGCCAATCCCTGGGTGAGTTTCACCAGTTTTGATTT
AAACGTGGCCAATATGGACAACTTCTTCGCCCCCGTTTTCACCATGGGCAAATATTATACGCAAGGCGACAAGGTGCTGATGCCGCTGGCGATTCA
*GGTTCATCATGCCGTTTGTGATGGCTTCCATGTCGGCAGAATGCTTAATGAATTACAACAGTACTGCGATGAGTGGCAGGGCGGGGCGTAA*TTTGA
TATCGAGCTCGCTTGGACTCCTGTTGATAGATC**CAGTAATGACCTCAGAACTCCAT**GTTTAACGGTAACGGTGTCCCGAAACGAACCCGTTTCGGG
ACAATTTCCAAAGTCTGTTCACTGGCATTAGCAACGGAAAATATTGTTCTGAATACGCTTCAGAACAAAACAGGTGCGGTTCCGACAGGAATA

CDS: *T7pol*
Locus tag: HO396_03645
GenBank ID: CP053602.1 DNA location: 751719-754370

Protein name:	T7 DNA-dependent RNA polymerase protein_id: QJZ11468.1
Nucleotide sequence:	***ATGAACACGATTAACATCGC****TAAGAACGACTTCTCTGACATCGAACTGGCTGCTATCCCGTTCAACACTCTGGCTGACCATTACGGTGAGCGTTTA*<br>*GCTCGCGAACAGTTGGCCCTTGAGCATGAGTCTTACGAGATGGGTGAAGCACGCTTCCGCAAGATGTTTGAGCGTCAACTTAAAGCTGGTGAGGTT*<br>*GCGGATAACGCTGCCGCCAAGCCTCTCATCACTACCCTACTCCCTAAGATGATTGCACGCATCAACGACTGGTTTGAGGAAGTGAAAGCTAAGCGC*<br>*GGCAAGCGCCCGACAGCCTTCCAGTTCCTGCAAGAAATCAAGCCGGAAGCCGTAGCGTACATCACCATTAAGACCACTCTGGCTTGCCTAACCAGT*<br>*GCTGACAATACAACCGTTCAGGCTGTAGCAAGCGCAATCGGTCGGGCCATTGAGGACGAGGCTCGCTTCGGTCGTATCCGTGACCTTGAAGCTAAG*<br>*CACTTCAAGAAAAACGTTGAGGAACAACTCAACAAGCGCGTAGGGCACGTCTACAAGAAAGCATTTATGCAAGTTGTCGAGGCTGACATGCTCTCT*<br>*AAGGGTCTACTCGGTGGCGAGGCGTGGTCTTCGTGGCATAAGGAAGACTCTATTCATGTAGGAGTACGCTGCATCGAGATGCTCATTGAGTCAACC*<br>*GGAATGGTTAGCTTACACCGCCAAAATGCTGGCGTAGTAGGTCAAGACTCTGAGACTATCGAACTCGCACCTGAATACGCTGAGGCTATCGCAACC*<br>*CGTGCAGGTGCGCTGGCTGGCATCTCTCCGATGTTCCAACCTTGCGTAGTTCCTCCTAAGCCGTGGACTGGCATTACTGGTGGTGGCTATTGGGCT*<br>*AACGGTCGTCGTCCTCTGGCGCTGGTGCGTACTCACAGTAAGAAAGCACTGATGCGCTACGAAGACGTTTACATGCCTGAGGTGTACAAAGCGATT*<br>*AACATTGCGCAAAACACCGCATGGAAAATCAACAAGAAAGTCCTAGCGGTCGCCAACGTAATCACCAAGTGGAAGCATTGTCCGGTCGAGGACATC*<br>*CCTGCGATTGAGCGTGAAGAACTCCCGATGAAACCGGAAGACATCGACATGAATCCTGAGGCTCTCACCGCGTGGAAACGTGCTGCCGCTGCTGTG*<br>*TACCGCAAGGACAAGGCTCGCAAGTCTCGCCGTATCAGCCTTGAGTTCATGCTTGAGCAAGCCAATAAGTTTGCTAACCATAAGGCCATCTGGTTC*<br>*CCTTACAACATGGACTGGCGCGGTCGTGTTTACGCTGTGTCAATGTTCAACCCGCAAGGTAACGATATGACCAAAGGACTGCTTACGCTGGCGAAA*<br>*GGTAAACCAATCGGTAAGGAAGGTTACTACTGGCTGAAAATCCACGGTGCAAACTGTGCGGGTGTCGATAAGGTTCCGTTCCCTGAGCGCATCAAG*<br>*TTCATTGAGGAAAACCACGAGAACATCATGGCTTGCGCTAAGTCTCCACTGGAGAACACTTGGTGGGCTGAGCAAGATTCTCCGTTCTGCTTCCTT*<br>*GCGTTCTGCTTTGAGTACGCTGGGGTACAGCACCACGGCCTGAGCTATAACTGCTCCCTTCCGCTGGCGTTTGACGGGTCTTGCTCTGGCATCCAG*<br>*CACTTCTCCGCGATGCTCCGAGATGAGGTAGGTGGTCGCGCGGTTAACTTGCTTCCTAGTGAAACCGTTCAGGACATCTACGGGATTGTTGCTAAG*<br>*AAAGTCAACGAGATTCTACAAGCAGACGCAATCAATGGGACCGATAACGAAGTAGTTACCGTGACCGATGAGAACACTGGTGAAATCTCTGAGAAA*<br>*GTCAAGCTGGGCACTAAGGCACTGGCTGGTCAATGGCTGGCTTACGGTGTTACTCGCAGTGTGACTAAGCGTTCAGTCATGACGCTGGCTTACGGG*<br>*TCCAAAGAGTTCGGCTTCCGTCAACAAGTGCTGGAAGATACCATTCAGCCAGCTATTGATTCCGGCAAGGGTCTGATGTTCACTCAGCCGAATCAG*<br>*GCTGCTGGATACATGGCTAAGCTGATTTGGGAATCTGTGAGCGTGACGGTGGTAGCTGCGGTTGAAGCAATGAACTGGCTTAAGTCTGCTGCTAAG*<br>*CTGCTGGCTGCTGAGGTCAAAGATAAGAAGACTGGAGAGATTCTTCGCAAGCGTTGCGCTGTGCATTGGGTAACTCCTGATGGTTTCCCTGTGTGG*<br>*CAGGAATACAAGAAGCCTATTCAGACGCGCTTGAACCTGATGTTCCTCGGTCAGTTCCGCTTACAGCCTACCATTAACACCAACAAAGATAGCGAG*<br>*ATTGATGCACACAAACAGGAGTCTGGTATCGCTCCTAACTTTGTACACAGCCAAGACGGTAGCCACCTTCGTAAGACTGTAGTGTGGGCACACGAG*<br>*AAGTACGGAATCGAATCTTTTGCACTGATTCACGACTCCTTCGGTACCATTCCGGCTGACGCTGCGAACCTGTTCAAAGCAGTGCGCGAAACTATG*<br>*GTTGACACATATGAGTCTTGTGATGTACTGGCTGATTTCTACGACCAGTTCGCTGACCAGTTGCACGAGTCTCAATTGGACAAAATGCCAGCACTT*<br>*CCGGCTAAAGGTAACTTGAACCTCCGTGACATCTTAGAGTCGGACTTCGCGTTCGCGTAA*

Protein sequence:	MNTINIAKNDFSDIELAAIPFNTLADHYGERLAREQLALEHESYEMGEARFRKMFERQLKAGEVADNAAAKPLITTLLPKMIARINDWFEEVKAKR<br>GKRPTAFQFLQEIKPEAVAYITIKTTLACLTSADNTTVQAVASAIGRAIEDEARFGRIRDLEAKHFKKNVEEQLNKRVGHVYKKAFMQVVEADMLS<br>KGLLGGEAWSSWHKEDSIHVGVRCIEMLIESTGMVSLHRQNAGVVGQDSETIELAPEYAEAIATRAGALAGISPMFQPCVVPPKPWTGITGGGYWA<br>NGRRPLALVRTHSKKALMRYEDVYMPEVYKAINIAQNTAWKINKKVLAVANVITKWKHCPVEDIPAIEREELPMKPEDIDMNPEALTAWKRAAAAV<br>YRKDKARKSRRISLEFMLEQANKFANHKAIWFPYNMDWRGRVYAVSMFNPQGNDMTKGLLTLAKGKPIGKEGYYWLKIHGANCAGVDKVPFPERIK<br>FIEENHENIMACAKSPLENTWWAEQDSPFCFLAFCFEYAGVQHHGLSYNCSLPLAFDGSCSGIQHFSAMLRDEVGGRAVNLLPSETVQDIYGIVAK<br>KVNEILQADAINGTDNEVVTVTDENTGEISEKVKLGTKALAGQWLAYGVTRSVTKRSVMTLAYGSKEFGFRQQVLEDTIQPAIDSGKGLMFTQPNQ<br>AAGYMAKLIWESVSVTVVAAVEAMNWLKSAAKLLAAEVKDKKTGEILRKRCAVHWVTPDGFPVWQEYKKPIQTRLNLMFLGQFRLQPTINTNKDSE<br>IDAHKQESGIAPNFVHSQDGSHLRKTVVWAHEKYGIESFALIHDSFGTIPADAANLFKAVRETMVDTYESCDVLADFYDQFADQLHESQLDKMPAL<br>PAKGNLNLRDILESDFAFA

CDS: *cat*
Locus tag: BWL12_13190
GenBank ID: CP019629.1 DNA location: 2468449-2469108

Protein name:	type A-1 chloramphenicol O-acetyltransferase protein_id: AQP92267.1
Nucleotide sequence:	*ATGGAGAAAAAAATCACTGGATATACCACCGTTGATATATCCCAATGGCATCGTAAAGAACATTTTGAGGCATTTCAGTCAGTTGCTCAATGTACC<br>TATAACCAGACCGTTCAGCTGGATATTACGGCCTTTTTAAAGACCGTAAAGAAAAATAAGCACAAGTTTTATCCGGCCTTTATTCACATTCTTGCC<br>CGCCTGATGAATGCTCATCCGGAATTCCGTATGGCAATGAAAGACGGTGAGCTGGTGATATGGGATAGTGTTCACCCTTGTTACACCGTTTTCCAT<br>GAGCAAACTGAAACGTTTTCATCGCTCTGGAGTGAATACCACGACGATTTCCGGCAGTTTCTACACATATATTCGCAAGATGTGGCGTGTTACGGT<br>GAAAACCTGGCCTATTTCCCTAAAGGGTTTATTGAGAATATGTTTTTCGTCTCAGCCAATCCCTGGGTGAGTTTCACCAGTTTTGATTTAAACGTG<br>GCCAATATGGACAACTTCTTCGCCCCCGTTTTCACCATGGGCAAATATTATACGCAAGGCGACAAGGTGCTGATGCCGCTGGCGATTCAGGTTCAT<br>CATGCCGTTTGTGATGGCTTCCATGTCGGCAGAATGCTTAATGAATTACAACAGTACTGCGATGAGTGGCAGGGCGGGGCGTAA*
Protein sequence:	MEKKITGYTTVDISQWHRKEHFEAFQSVAQCTYNQTVQLDITAFLKTVKKNKHKFYPAFIHILARLMNAHPEFRMAMKDGELVIWDSVHPCYTVFH<br>EQTETFSSLWSEYHDDFRQFLHIYSQDVACYGENLAYFPKGFIENMFFVSANPWVSFTSFDLNVANMDNFFAPVFTMGKYYTQGDKVLMPLAIQVH<br>HAVCDGFHVGRMLNELQQYCDEWQGGA

Sequencing results

from: OutSeqGad FW Genes: *T7pol* Primers: gadBt7 FW

TCCGAAGCGTCCAAGAAACTGCAGGGGTATTGCCCAACAGTAACAGCTTTAAACATACCTGATAAC**AGGAGGTAAATA*ATGCACACGATTAACATC***
***GC**TAAGAACGACTTCTCTGACATCGAACTGGCTGCTATCCCGTTCAACACTCTGGCTGACCATTACGGTGAGCGTTTAGCTCGCGAACAGTTGGCC*
CTTGAGCATGAGTCTTACGAGATGGGTGAAGCACGCTTCCGCAAGATGTTTGAGCGTCAACTTAAAGCTGGTGAGGTTGCGGATAACGCTGCCGCC
AAGCCTCTCATCACTACCCTACTCCCTAAGATGATTGCACGCATCAACGACTGGTTTGAGGAAGTGAAAGCTAAGCGCGGCAAGCGCCCGACAGCC
TTCCAGTTCCTGCAAGAAATCAAGCCGGAAGCCGTAGCGTACATCACCATTAAGACCACTCTGGCTTGCCTAACCAGTGCTGACAATACAACCGTT
CAGGCTGTAGCAAGCGCAATCGGTCGGGCCATTGAGGACGAGGCTCGCTTCGGTCGTATCCGTGACCTTGAAGCTAAGCACTTCAAGAAAAACGTT
GAGGAACAACTCAACAAGCGCGTAGGGCACGTCTACAAGAAAGCATTTATGCAAGTTGTCGAGGCTGACATGCTCTCTAAGGGTCTACTCGGTGGC
GAGGCGTGGTCTTCGTGGCATAAGGAAGACTCTATTCATGTAGGAGTACGCTGCATCGAGATGCTCATTGAGTCAACCGGAATGGTTAGCTTACAC
CGCCAAAATGCTGGCGTAGTAGGTCAAGACTCTGAGACTATCGAACTCGCACCTGAATACGCTGAGGCTATCGCAACCCGTGCAGGTGCGCTGGCT
GGCATCTCTCCGATGTTCCAACCTTGCGTAGTTCCTCCTAAGCCGTGGACTGGCATTACTGGTGGTGGCTATTGGGCTAACGGTCGTCGTCCTCTG
GCGCTGGTGCGTACTCACAGTAAGAAAGCACTGATGCGCTACGAAGACGTTTACATGCCTGAGGTGTACAAAGCGATTAACATTGCGCAAAACACC
GCATGGAAAATCAACAAGAAAGTCCTAGCGGTCGCCAACGTAATCACCAAGTGGAAGCATTGGCGCCGCGAGGACATCCCTGCGATTGAACGTGAA
GAACTCCCGATGAAACCGGAAGACATCGACATGAATCCTGAGGCTTTCACCGCGTGGAAACCTGCTGCCGCTGCTGGTACCGCAAGACAAGGCTCC
*AAGTTTCGCCTATCACCCTTGATTCATGCTTGACAAGCCAATAGTTTGCTAACCTAAGGCCTTTGGTCCCTTAAAAGGAAGGGCGGGTCGGGTT*CC
CTTGTTATTTTTCACCCCCAGGAACTATTAACCAAGGGATTTTCCTGGGAAAGAACAACGGAAAGAAGGTTCTCGGTAAAACCCCGGGGAAAATTG
CGGGTGGTAAAAGTTCTTCCGGCCCATATTTTAGAACACAAAAAATTGGGTTCTATCCCGGAAAAATGGGGGGAAATCCCCTTTTTTTTTTTTTAA
GGGGACCCACCGATATTTCCCCTGTGGGTTCTTCCCTCCCCCAAGGGGGGGCTTTTTAAACCAAATTTAAAAAAAACAAAAAAAAAAAAAAAAAGG
GGTGTTGTTTTATTTAATTTGTATTCTCTCATTTCCCCACACACTTAATTAGGCGGCGAGGGTGGGGC

Alignments: against *T7pol*

Query: sequencing data Sbjct(subject): *T7pol*

Score	Expect	Identities	Gaps	Strand
2194 bits(1188)	0.0	1252/1280(98%)	16/1280(1%)	Plus/Plus

```
Query  78      AATGCACACGATTAACATCGCTAAGAACGACTTCTCTGACATCGAACTGGCTGCTATCCC  137
               |||| |||||||||||||||||||||||||||||||||||||||||||||||||||||||
Sbjct  751718  AATGAACACGATTAACATCGCTAAGAACGACTTCTCTGACATCGAACTGGCTGCTATCCC  751777

Query  138     GTTCAACACTCTGGCTGACCATTACGGTGAGCGTTTAGCTCGCGAACAGTTGGCCCTTGA  197
               ||||||||||||||||||||||||||||||||||||||||||||||||||||||||||||
Sbjct  751778  GTTCAACACTCTGGCTGACCATTACGGTGAGCGTTTAGCTCGCGAACAGTTGGCCCTTGA  751837

Query  198     GCATGAGTCTTACGAGATGGGTGAAGCACGCTTCCGCAAGATGTTTGAGCGTCAACTTAA  257
               ||||||||||||||||||||||||||||||||||||||||||||||||||||||||||||
Sbjct  751838  GCATGAGTCTTACGAGATGGGTGAAGCACGCTTCCGCAAGATGTTTGAGCGTCAACTTAA  751897

Query  258     AGCTGGTGAGGTTGCGGATAACGCTGCCGCCAAGCCTCTCATCACTACCCTACTCCCTAA  317
               ||||||||||||||||||||||||||||||||||||||||||||||||||||||||||||
Sbjct  751898  AGCTGGTGAGGTTGCGGATAACGCTGCCGCCAAGCCTCTCATCACTACCCTACTCCCTAA  751957

Query  318     GATGATTGCACGCATCAACGACTGGTTTGAGGAAGTGAAAGCTAAGCGCGGCAAGCGCCC  377
               ||||||||||||||||||||||||||||||||||||||||||||||||||||||||||||
Sbjct  751958  GATGATTGCACGCATCAACGACTGGTTTGAGGAAGTGAAAGCTAAGCGCGGCAAGCGCCC  752017
```

```
Query  378     GACAGCCTTCCAGTTCCTGCAAGAAATCAAGCCGGAAGCCGTAGCGTACATCACCATTAA  437
               ||||||||||||||||||||||||||||||||||||||||||||||||||||||||||||
Sbjct  752018  GACAGCCTTCCAGTTCCTGCAAGAAATCAAGCCGGAAGCCGTAGCGTACATCACCATTAA  752077

Query  438     GACCACTCTGGCTTGCCTAACCAGTGCTGACAATACAACCGTTCAGGCTGTAGCAAGCGC  497
               ||||||||||||||||||||||||||||||||||||||||||||||||||||||||||||
Sbjct  752078  GACCACTCTGGCTTGCCTAACCAGTGCTGACAATACAACCGTTCAGGCTGTAGCAAGCGC  752137

Query  498     AATCGGTCGGGCCATTGAGGACGAGGCTCGCTTCGGTCGTATCCGTGACCTTGAAGCTAA  557
               ||||||||||||||||||||||||||||||||||||||||||||||||||||||||||||
Sbjct  752138  AATCGGTCGGGCCATTGAGGACGAGGCTCGCTTCGGTCGTATCCGTGACCTTGAAGCTAA  752197

Query  558     GCACTTCAAGAAAAACGTTGAGGAACAACTCAACAAGCGCGTAGGGCACGTCTACAAGAA  617
               ||||||||||||||||||||||||||||||||||||||||||||||||||||||||||||
Sbjct  752198  GCACTTCAAGAAAAACGTTGAGGAACAACTCAACAAGCGCGTAGGGCACGTCTACAAGAA  752257

Query  618     AGCATTTATGCAAGTTGTCGAGGCTGACATGCTCTCTAAGGGTCTACTCGGTGGCGAGGC  677
               ||||||||||||||||||||||||||||||||||||||||||||||||||||||||||||
Sbjct  752258  AGCATTTATGCAAGTTGTCGAGGCTGACATGCTCTCTAAGGGTCTACTCGGTGGCGAGGC  752317

Query  678     GTGGTCTTCGTGGCATAAGGAAGACTCTATTCATGTAGGAGTACGCTGCATCGAGATGCT  737
               ||||||||||||||||||||||||||||||||||||||||||||||||||||||||||||
Sbjct  752318  GTGGTCTTCGTGGCATAAGGAAGACTCTATTCATGTAGGAGTACGCTGCATCGAGATGCT  752377

Query  738     CATTGAGTCAACCGGAATGGTTAGCTTACACCGCCAAAATGCTGGCGTAGTAGGTCAAGA  797
               ||||||||||||||||||||||||||||||||||||||||||||||||||||||||||||
Sbjct  752378  CATTGAGTCAACCGGAATGGTTAGCTTACACCGCCAAAATGCTGGCGTAGTAGGTCAAGA  752437

Query  798     CTCTGAGACTATCGAACTCGCACCTGAATACGCTGAGGCTATCGCAACCCGTGCAGGTGC  857
               ||||||||||||||||||||||||||||||||||||||||||||||||||||||||||||
Sbjct  752438  CTCTGAGACTATCGAACTCGCACCTGAATACGCTGAGGCTATCGCAACCCGTGCAGGTGC  752497

Query  858     GCTGGCTGGCATCTCTCCGATGTTCCAACCTTGCGTAGTTCCTCCTAAGCCGTGGACTGG  917
               ||||||||||||||||||||||||||||||||||||||||||||||||||||||||||||
Sbjct  752498  GCTGGCTGGCATCTCTCCGATGTTCCAACCTTGCGTAGTTCCTCCTAAGCCGTGGACTGG  752557

Query  918     CATTACTGGTGGTGGCTATTGGGCTAACGGTCGTCGTCCTCTGGCGCTGGTGCGTACTCA  977
               ||||||||||||||||||||||||||||||||||||||||||||||||||||||||||||
Sbjct  752558  CATTACTGGTGGTGGCTATTGGGCTAACGGTCGTCGTCCTCTGGCGCTGGTGCGTACTCA  752617

Query  978     CAGTAAGAAAGCACTGATGCGCTACGAAGACGTTTACATGCCTGAGGTGTACAAAGCGAT  1037
               ||||||||||||||||||||||||||||||||||||||||||||||||||||||||||||
Sbjct  752618  CAGTAAGAAAGCACTGATGCGCTACGAAGACGTTTACATGCCTGAGGTGTACAAAGCGAT  752677
```

```
Query  1038    TAACATTGCGCAAAACACCGCATGGAAAATCAACAAGAAAGTCCTAGCGGTCGCCAACGT  1097
               ||||||||||||||||||||||||||||||||||||||||||||||||||||||||||||
Sbjct  752678  TAACATTGCGCAAAACACCGCATGGAAAATCAACAAGAAAGTCCTAGCGGTCGCCAACGT  752737

Query  1098    AATCACCAAGTGGAAGCATTGGCGCCG-CGAGGACATCCCTGCGATTGAACGTGAAGAAC  1156
               ||||||||||||||||||||| | | | ||||||||||||||||||||| ||||||||||
Sbjct  752738  AATCACCAAGTGGAAGCATTGTC-CGGTCGAGGACATCCCTGCGATTGAGCGTGAAGAAC  752796

Query  1157    TCCCGATGAAACCGGAAGACATCGACATGAATCCTGAGGCTTTCACCGCGTGGAAACCTG  1216
               ||||||||||||||||||||||||||||||||||||||||| ||||||||||||||| ||
Sbjct  752797  TCCCGATGAAACCGGAAGACATCGACATGAATCCTGAGGCTCTCACCGCGTGGAAACGTG  752856

Query  1217    CTGCCGCTGCTG-GTACCGCAAG-ACAAGGCTC-CAAGTTTCGCC-TATCACCCTTGA-T  1271
               |||||||||||| |||||||||| ||||||||| ||||| ||||| ||||| |||||| |
Sbjct  752857  CTGCCGCTGCTGTGTACCGCAAGGACAAGGCTCGCAAGTCTCGCCGTATCAGCCTTGAGT  752916

Query  1272    TCATGCTTGA-CAAGCCAATA-GTTTGCTAACC-TAAGGCCTT-TGGT-CCCTTA-AA-A  1324
               |||||||||| |||||||||| ||||||||||| ||||||| | |||| |||||| || |
Sbjct  752917  TCATGCTTGAGCAAGCCAATAAGTTTGCTAACCATAAGGCCATCTGGTTCCCTTACAACA  752976

Query  1325    -GGAAGGGCG-GGTCGGGTT  1342
                |||  |||| ||||| |||
Sbjct  752977  TGGACTGGCGCGGTCGTGTT  752996
```

from: OutSeqGad RV Genes: *cat* Primers: gadBt7 RV

AAAGGGTGACGTAATCAGAACATATTTTCCGTTGCTAATGCCAGTGAACAGACTTTGGAAATTGTCCCGAAACGGGTTCGTTTCGGACACCGTTAC
CGTTAAAC**ATGGAGTTCTGAGGTCATTACTG**GATCTATCAACAGGAGTCCAAGCGAGCTCGATATCAAA*TTACGCCCCGCCCTGCCACTCATCGCA*
GTACTGTTGTAATTCATTAAGCATTCTGCCGACATGGAAGCCATCACAAACGGCATGATGAACCTGAATCGCCAGCGGCATCAGCACCTTGTCGCC
TTGCGTATAATATTTGCCCATGGTGAAAACGGGGGCGAAGAAGTTGTCCATATTGGCCACGTTTAAATCAAAACTGGTGAAACTCACCCAGGGATT
GGCTGAGACGAAAAACATATTCTCAATAAACCCTTTAGGGAAATAGGCCAGGTTTTCACCGTAACACGCCACATCTTGCGAATATATGTGTAGAAA
CTGCCGGAAATCGTCGTGGTATTCACTCCAGAGCGATGAAAACGTTTCAGTTTGCTCATGGAAAACGGTGTAACAAGGGTGAACACTATCCCATAT
CACCAGCTCACCGTCTTTCATTGCCATACGGAATTCCGGATGAGCATTCATCAGGCGGGCAAGAATGTGAATAAAGGCCGGATAAAACTTGTGCTT
ATTTTTCTTTACGGTCTTTAAAAAGGCCGTAATATCCAGCTGAACGGTCTGGTTATAGGTACATTGAGCAACTGACTGAAATGCCTCAAAATGTTC
*TTTACGATGCCATTGGGATATATCAACGGTGGTATATCCAGTGATTTTTTTCTCCAT*TTTAGCTTCCTTAGCTCCTGAAAATCTCGATAACTCAAA
AAATACGCCCGGTAGTGATCTTATTTCATTATGGTGAAAGTTGGAACCTCTTACGTGCCGATCAACGTCTCATTTTCGCCAGATATGGGCCC*TTAC*
GCGAACGCGAAGTCCGACTCTAAGATGTCACGGAGGTTCAAGTTACCTTTAGCCGGAAGTGCTGGCATTTTGTCCAATTGAGACTCGTGCAACTGG
TCAGCGAACTGGTCGTAGAAATCAGCCAGTACATCACAAGACTCATATGTGTCACACTTAGTTTCGCGCACTGCTTTGAACAGGTTCGCACCGTCA
GCCGAATGGTACCAAAGAATCGTGAATCAATGCAAAAGATTCGATTCCGTACTTCTCGGTGCCCACTACGGTCTTACAAAGGGGCTACCGTCTGGC
*TGGTACAAGTAGAACGAACCCGAATCCGGTTGGGGCTCATCCGCTTTCTTGTTGGGTTAAGGAAGCTTTAA*CGAAATACCCAAAAATCGTTAACCC
TTTAAAAGTTTTGTTTTCCCACGGAAACTTAGATTCCGGGCACCCCCTTGGAAAATTTCTTTTTTTTACACTAATCCAATGTACTGACTACCTTTT
AGAGTTTTTATTTCTTGATTCAGTTTAATTTATTACCATCTCTCTATATACAAGGATGTGAATATATGTTGCATGTTAGGTGAGCTTTTTTGTTAT

TGTAGTATTTTGTTGGGGAGTGCTAGGCCATCTCGTATTTATTCAATCCATTACTTTTATATATCTATTATTATTTATTGCGTGAACAGGATTTAC
CCAAAATATATATATCATGATT

Reverse complement:

AATCATGATATATATATTTTGGGTAAATCCTGTTCACGCAATAAATAATAATAGATATATAAAAGTAATGGATTGAATAAATACGAGATGGCCTAG
CACTCCCCAACAAAATACTACAATAACAAAAAAGCTCACCTAACATGCAACATATATTCACATCCTTGTATATAGAGAGATGGTAATAAATTAAAC
TGAATCAAGAAATAAAAACTCTAAAAGGTAGTCAGTACATTGGATTAGTGTAAAAAAAAGAAATTTTCCAAGGGGGTGCCCGGAATCTAAGTTTCC
GTGGGAAAACAAAACTTTTAAAGGGTTAACGATTTTTGGGTATTTCG*TTAAAGCTTCCTTAACCCAACAAGAAAGCGGATGAGCCCCAACCGGATT*
CGGGTTCGTTCTACTTGTACCAGCCAGACGGTAGCCCCTTTGTAAGACCGTAGTGGGCACCGAGAAGTACGGAATCGAATCTTTTGCATTGATTCA
CGATTCTTTGGTACCATTCGGCTGACGGTGCGAACCTGTTCAAAGCAGTGCGCGAAACTAAGTGTGACACATATGAGTCTTGTGATGTACTGGCTG
ATTTCTACGACCAGTTCGCTGACCAGTTGCACGAGTCTCAATTGGACAAAATGCCAGCACTTCCGGCTAAAGGTAACTTGAACCTCCGTGACATCT
*TAGAGTCGGACTTCGCGTTCGCGTAA*GGGCCCATATCTGGCGAAAATGAGACGTTGATCGGCACGTAAGAGGTTCCAACTTTCACCATAATGAAAT
AAGATCACTACCGGGCGTATTTTTTGAGTTATCGAGATTTTCAGGAGCTAAGGAAGCTAAA*ATGGAGAAAAAAATCACTGGATATACCACCGTTGA*
TATATCCCAATGGCATCGTAAAGAACATTTTGAGGCATTTCAGTCAGTTGCTCAATGTACCTATAACCAGACCGTTCAGCTGGATATTACGGCCTT
TTTAAAGACCGTAAAGAAAAATAAGCACAAGTTTTATCCGGCCTTTATTCACATTCTTGCCCGCCTGATGAATGCTCATCCGGAATTCCGTATGGC
AATGAAAGACGGTGAGCTGGTGATATGGGATAGTGTTCACCCTTGTTACACCGTTTTCCATGAGCAAACTGAAACGTTTTCATCGCTCTGGAGTGA
ATACCACGACGATTTCCGGCAGTTTCTACACATATATTCGCAAGATGTGGCGTGTTACGGTGAAAACCTGGCCTATTTCCCTAAAGGGTTTATTGA
GAATATGTTTTTCGTCTCAGCCAATCCCTGGGTGAGTTTCACCAGTTTTGATTTAAACGTGGCCAATATGGACAACTTCTTCGCCCCCGTTTTCAC
CATGGGCAAATATTATACGCAAGGCGACAAGGTGCTGATGCCGCTGGCGATTCAGGTTCATCATGCCGTTTGTGATGGCTTCCATGTCGGCAGAAT
*GCTTAATGAATTACAACAGTACTGCGATGAGTGGCAGGGCGGGGCGTAA*TTTGATATCGAGCTCGCTTGGACTCCTGTTGATAGATC**CAGTAATGA**
CCTCAGAACTCCATGTTTAACGGTAACGGTGTCCGAAACGAACCCGTTTCGGGACAATTTCCAAAGTCTGTTCACTGGCATTAGCAACGGAAAATA
TGTTCTGATTACGTCACCCTTT

Alignments: against *cat*

Query: sequencing data Sbjct(subject): *cat*

```
Score            Expect      Identities     Gaps        Strand
1557 bits(843)   0.0         845/846(99%)   0/846(0%)   Plus/Minus

Query  105      ATGGAGTTCTGAGGTCATTACTGGATCTATCAACAGGAGTCCAAGCGAGCTCGATATCAA  164
                ||||||||||||||||||||||||||||||||||||||||||||||||||||||||||||
Sbjct  2469169  ATGGAGTTCTGAGGTCATTACTGGATCTATCAACAGGAGTCCAAGCGAGCTCGATATCAA  2469110

Query  165      ATTACGCCCCGCCCTGCCACTCATCGCAGTACTGTTGTAATTCATTAAGCATTCTGCCGA  224
                ||||||||||||||||||||||||||||||||||||||||||||||||||||||||||||
Sbjct  2469109  ATTACGCCCCGCCCTGCCACTCATCGCAGTACTGTTGTAATTCATTAAGCATTCTGCCGA  2469050

Query  225      CATGGAAGCCATCACAAACGGCATGATGAACCTGAATCGCCAGCGGCATCAGCACCTTGT  284
                ||||||||||||||||||||||||||||||||||||||||||||||||||||||||||||
Sbjct  2469049  CATGGAAGCCATCACAAACGGCATGATGAACCTGAATCGCCAGCGGCATCAGCACCTTGT  2468990

Query  285      CGCCTTGCGTATAATATTTGCCCATGGTGAAAACGGGGGCGAAGAAGTTGTCCATATTGG  344
                ||||||||||||||||||||||||||||||||||||||||||||||||||||||||||||
Sbjct  2468989  CGCCTTGCGTATAATATTTGCCCATGGTGAAAACGGGGGCGAAGAAGTTGTCCATATTGG  2468930

Query  345      CCACGTTTAAATCAAAACTGGTGAAACTCACCCAGGGATTGGCTGAGACGAAAAACATAT  404
```

```
                  ||||||||||||||||||||||||||||||||||||||||||||||||||||||||||||
Sbjct  2468929  CCACGTTTAAATCAAAACTGGTGAAACTCACCCAGGGATTGGCTGAGACGAAAAACATAT  2468870

Query  405      TCTCAATAAACCCTTTAGGGAAATAGGCCAGGTTTTCACCGTAACACGCCACATCTTGCG  464
                ||||||||||||||||||||||||||||||||||||||||||||||||||||||||||||
Sbjct  2468869  TCTCAATAAACCCTTTAGGGAAATAGGCCAGGTTTTCACCGTAACACGCCACATCTTGCG  2468810

Query  465      AATATATGTGTAGAAACTGCCGGAAATCGTCGTGGTATTCACTCCAGAGCGATGAAAACG  524
                |||||||||||||||||||||||||||||||||||||||| |||||||||||||||||||
Sbjct  2468809  AATATATGTGTAGAAACTGCCGGAAATCGTCGTGGTATTCGCTCCAGAGCGATGAAAACG  2468750

Query  525      TTTCAGTTTGCTCATGGAAAACGGTGTAACAAGGGTGAACACTATCCCATATCACCAGCT  584
                ||||||||||||||||||||||||||||||||||||||||||||||||||||||||||||
Sbjct  2468749  TTTCAGTTTGCTCATGGAAAACGGTGTAACAAGGGTGAACACTATCCCATATCACCAGCT  2468690

Query  585      CACCGTCTTTCATTGCCATACGGAATTCCGGATGAGCATTCATCAGGCGGGCAAGAATGT  644
                ||||||||||||||||||||||||||||||||||||||||||||||||||||||||||||
Sbjct  2468689  CACCGTCTTTCATTGCCATACGGAATTCCGGATGAGCATTCATCAGGCGGGCAAGAATGT  2468630

Query  645      GAATAAAGGCCGGATAAAACTTGTGCTTATTTTTCTTTACGGTCTTTAAAAAGGCCGTAA  704
                ||||||||||||||||||||||||||||||||||||||||||||||||||||||||||||
Sbjct  2468629  GAATAAAGGCCGGATAAAACTTGTGCTTATTTTTCTTTACGGTCTTTAAAAAGGCCGTAA  2468570

Query  705      TATCCAGCTGAACGGTCTGGTTATAGGTACATTGAGCAACTGACTGAAATGCCTCAAAAT  764
                ||||||||||||||||||||||||||||||||||||||||||||||||||||||||||||
Sbjct  2468569  TATCCAGCTGAACGGTCTGGTTATAGGTACATTGAGCAACTGACTGAAATGCCTCAAAAT  2468510

Query  765      GTTCTTTACGATGCCATTGGGATATATCAACGGTGGTATATCCAGTGAtttttttCTCCA  824
                ||||||||||||||||||||||||||||||||||||||||||||||||||||||||||||
Sbjct  2468509  GTTCTTTACGATGCCATTGGGATATATCAACGGTGGTATATCCAGTGATTTTTTTCTCCA  2468450

Query  825      TTTTAGCTTCCTTAGCTCCTGAAAATCTCGATAACTCAAAAAATACGCCCGGTAGTGATC  884
                ||||||||||||||||||||||||||||||||||||||||||||||||||||||||||||
Sbjct  2468449  TTTTAGCTTCCTTAGCTCCTGAAAATCTCGATAACTCAAAAAATACGCCCGGTAGTGATC  2468390

Query  885      TTATTTCATTATGGTGAAAGTTGGAACCTCTTACGTGCCGATCAACGTCTCATTTTCGCC  944
                ||||||||||||||||||||||||||||||||||||||||||||||||||||||||||||
Sbjct  2468389  TTATTTCATTATGGTGAAAGTTGGAACCTCTTACGTGCCGATCAACGTCTCATTTTCGCC  2468330

Query  945      AGATAT  950
                ||||||
Sbjct  2468329  AGATAT  2468324
```

```
Score              Expect     Identities      Gaps          Strand
161 bits(87)       7e-36      94/97(97%)      2/97(2%)      Plus/Plus

Query  10       CGTAATCAGAAC-ATATTTTCCGTTGCTAATGCCAGTGAACAGACTTTGGAAATTGTCCC  68
                |||| ||||||| |||||||||||||||||||||||||||||||||||||||||||||||
Sbjct  1570544  CGTATTCAGAACAATATTTTCCGTTGCTAATGCCAGTGAACAGACTTTGGAAATTGTCCC  1570603

Query  69       GAAACGGGTTCGTTTC-GGACACCGTTACCGTTAAAC  104
                |||||||||||||||| ||||||||||||||||||||
Sbjct  1570604  GAAACGGGTTCGTTTCGGGACACCGTTACCGTTAAAC  1570640
```

against *T7pol*:

```
Query: sequencing data Sbjct(subject): T7pol
Score              Expect     Identities      Gaps          Strand
479 bits(259)      1e-134     346/384(90%)    21/384(5%)    Plus/Minus
Query  957     TTACGCGAACGCGAAGTCCGACTCTAAGATGTCACGGAGGTTCAAGTTACCTTTAGCCGG  1016
               ||||||||||||||||||||||||||||||||||||||||||||||||||||||||||||
Sbjct  754370  TTACGCGAACGCGAAGTCCGACTCTAAGATGTCACGGAGGTTCAAGTTACCTTTAGCCGG  754311

Query  1017    AAGTGCTGGCATTTTGTCCAATTGAGACTCGTGCAACTGGTCAGCGAACTGGTCGTAGAA  1076
               ||||||||||||||||||||||||||||||||||||||||||||||||||||||||||||
Sbjct  754310  AAGTGCTGGCATTTTGTCCAATTGAGACTCGTGCAACTGGTCAGCGAACTGGTCGTAGAA  754251

Query  1077    ATCAGCCAGTACATCACAAGACTCATATGTGTCACACTTAGTTTCGCGCACTGCTTTGAA  1136
               ||||||||||||||||||||||||||||||||||  | ||||||||||||||||||||||
Sbjct  754250  ATCAGCCAGTACATCACAAGACTCATATGTGTCAACCATAGTTTCGCGCACTGCTTTGAA  754191

Query  1137    CAGGTTCGCACCGTCAGCCG-AATGGTACCAAAGAA-TCGTGAATCAATGCAAAAGATTC  1194
               |||||||||| ||||||||| ||||||||| ||| | |||||||||| ||||||||||||
Sbjct  754190  CAGGTTCGCAGCGTCAGCCGGAATGGTACCGAAGGAGTCGTGAATCAGTGCAAAAGATTC  754131

Query  1195    GATTCCGTACTTCTCG-GTGCC--CACTACGGTCTTACAAAGG-GGCTACCGTCT-GGCT  1249
               |||||||||||||||| |||||  |||||| ||||||| |||| ||||||||||| ||||
Sbjct  754130  GATTCCGTACTTCTCGTGTGCCCACACTACAGTCTTACGAAGGTGGCTACCGTCTTGGCT  754071

Query  1250    G-GTACAA-GT-AGAA-CGA-ACCCGAATCCGGTTGG-G-GC-TCA-TC-CGCTTTCTT-  1298
               | |||||| || || | ||| ||| || ||| ||| | | || ||| || |||| |||| 
Sbjct  754070  GTGTACAAAGTTAGGAGCGATACCAGACTCCTGTTTGTGTGCATCAATCTCGCTATCTTT  754011

Query  1299    GTTGG-GTTAA-GGAAG-CTTTAA  1319
               ||||| ||||| || || || |||
Sbjct  754010  GTTGGTGTTAATGGTAGGCTGTAA  753987
```
